# Integrated Genomics and Phenotypic Analysis of *Pediococcus pentosaceus* BGI-N8 and *Pediococcus acidilactici* BGI-N9: Partial Evidence Suggesting In Vitro Probiotic Properties to Glycolipid Metabolism Regulation Potential

**DOI:** 10.3390/microorganisms14081614

**Published:** 2026-07-24

**Authors:** Jiayi Ma, Zhihui Ma, Xinyu Yang, Yuanyuan Yao, Benliang Wei, Jielei Zhu, Qiang Luo, Haifeng Zhang, Liang Xiao, Yiyi Zhong, Yuanqiang Zou

**Affiliations:** 1 State Key Laboratory of Genome and Multi-Omics Technologies, BGI Research, Shenzhen 518083, China; majiayi@genomics.cn (J.M.); yangxinyu2@genomics.cn (X.Y.); zhujielei@genomics.cn (J.Z.); 2 College of Life Sciences, University of Chinese Academy of Sciences, Beijing 100049, China; 3 BGI Precision Nutrition, Shenzhen 518083, China; mazhihui@genomics.cn (Z.M.); weibenliang@genomics.cn (B.W.); luoqiang@genomics.cn (Q.L.); zhanghaifeng@genomics.cn (H.Z.); xiaoliang@genomics.cn (L.X.); 4 BGI Research, Shenzhen 518083, China; 19231256051@163.com; 5 Shenzhen Key Laboratory of Human Commensal Microorganisms and Health Research, BGI Research, Shenzhen 518083, China

**Keywords:** *Pediococcus*, whole-genome sequencing, probiotics, α-glucosidase inhibition, cholesterol lowering

## Abstract

Dietary interventions using probiotics actively regulate metabolism in obesity and type 2 diabetes. This study aimed to characterize two novel co-isolated strains, *P. pentosaceus* BGI-N8 and *P. acidilactici* BGI-N9, using an integrated genome–phenotype approach. Whole-genome sequencing established their preliminary safety and functional genotypes, followed by in vitro assays measuring gastrointestinal tolerance, adhesion, and metabolic enzyme activity. Genomic analysis revealed that both strains achieved genome-based safety levels, and no high-confidence transferable AMR determinants and canonical virulence factors were identified. Functional annotation showed that BGI-N8 and BGI-N9 contain essential genes for gastrointestinal adaptation, including F0F1-ATPase, the *dltA-D* operon, and the *opp* transport system. Phenotypically, BGI-N9 exhibited superior acid resistance (79.3% survival at pH 2.0), while both strains showed robust auto-aggregation (>70%) and high adhesion to HT-29 cells (ranged from 5.4 to 5.9 adherent bacteria per cell). Furthermore, the strains displayed inhibitory activity against selected indicator pathogens (88.6–97.6% inhibition). Notably, BGI-N8 and BGI-N9 showed comparable α-glucosidase inhibitory activities, with inhibition rates of 44.53% and 39.94%, respectively, Both strains also exhibited comparable cholesterol-removal capacities under the tested conditions, with removal rates of 68.76% and 74.49%. Overall, these genomic and phenotypic results supported the potentials of BGI-N8 and BGI-N9 as candidate probiotic strains with distinct complementary strengths in glycolipid regulation, providing a theoretical basis for their synergistic application. Further mechanistic, safety, and in vivo studies are required to validate their metabolic-health-related relevance.

## 1. Introduction

Lactic acid bacteria (LAB) are widely recognized for their probiotic benefits, particularly in promoting gut homeostasis and exerting positive regulatory effects on host metabolism. Traditional fermented foods serve as vital microbial reservoirs for such organisms, where natural selection under environmental pressures fosters the evolution of robust stress tolerance and specialized health-promoting attributes [1]. Among these, the genus *Pediococcus* has emerged as a potent candidate for functional applications. For example, *Pediococcus* strains exert cholesterol-lowering effects via bile salt hydrolase (bsh) activity, which deconjugates bile acids and forces hepatic cholesterol consumption for de novo synthesis [2]. Furthermore, clinical and in vivo studies on *P. pentosaceus* and *P. acidilactici* have demonstrated significant reductions in body weight, fasting blood glucose, and pro-inflammatory cytokines (e.g., TNF-α and IL-6) by modulating host signaling pathways [3]. These metabolic benefits are further supported by their secretion of bacteriocins: ribosomally synthesized peptides that preserve gut homeostasis by disrupting the membranes of pathogenic competitors, thereby preventing the gut dysbiosis often linked to metabolic dysfunction [4].

However, probiotic efficacy remains highly strain-specific, and current research has critical limitations that hinder the precise characterization and screening of probiotic candidates, although several studies have characterized individual *Pediococcus* strains with probiotic potential [5], or compare strains from geographically and ecologically distinct niches [6]. To date, comparative genomic and phenotypic analyses of co-isolated *Pediococcus* species originating from the same dairy microenvironment remain limited. Such co-isolated strains that have evolved within the same microenvironment offer a unique scientific advantage, as they minimize environmental interference and allow for a more precise comparison of inherent genomic and functional divergences [7]. Simultaneously, the advent of high-throughput sequencing has revolutionized probiotic screening methodologies, moving beyond time-consuming phenotypic assays to an efficient genomic-phenotypic strategy [8]. Whole-genome sequencing (WGS) enables comprehensive safety assessment such as detecting transferable antibiotic resistance genes or virulence factors and predicts key functions including gastrointestinal stress tolerance, which refers to survival under gastric acid, bile salt, and intestinal fluid exposure and bioactive compound synthesis, which refers to the potential production of functional metabolites or enzymes. These predictions can be further evaluated using targeted in vitro assays to form a robust candidate selection framework [9]. Our findings evaluate the specific probiotic potential of these strains and provide a replicable methodological framework for the integrative characterization of co-isolated *Pediococcus* strains.

*P. pentosaceus* BGI-N8 and *P. acidilactici* BGI-N9 were co-isolated from the same Inner Mongolian fermented yogurt microenvironment during our preliminary screening of *lactic* acid bacteria. Both strains showed stable growth under laboratory culture conditions and favorable preliminary fermentation-related characteristics, including biomass accumulation and acidification performance, and represented two different *Pediococcus* species from the same fermentation niche, providing a suitable comparative pair for genome-guided evaluation of strain-specific probiotic candidate traits. Their complete genomic sequences are archived in the Cultivated Genome Reference (CGR2) database [10] and maintained in its corresponding physical strain library. We mapped the genetic basis of their probiotic traits and tested these genomic models through targeted in vitro assays. This approach indicates their probiotic potential to regulate host metabolism and establishes a replicable framework for characterizing co-isolated *Pediococcus* strains.

## 2. Materials and Methods

### 2.1. Bacterial Strains and Cell Line

All the bacterial strains and the cell line used in the experiments were obtained from BGI Precision Nutrition, Shenzhen, China. BGI-N8 and BGI-N9 and *Lacticaseibacillus paracasei* ATCC 334 were cultured in De Man, Rogosa & Sharpe (MRS, Huankai Microbial Technology, Guangzhou, China) medium at 37 °C for 24 h, and were subcultured twice for activation. Four pathogenic strains, including *Escherichia coli* ATCC 25922, *Staphylococcus aureus* ATCC 29213, *Pseudomonas aeruginosa* ATCC 9027 and *Enterobacter cloacae* ATCC 23355 were cultured in Brain Heart Infusion (BHI, Aobox Biotechnology, Beijing, China) medium at 37 °C for 24 h. Human colon cancer cell line HT-29 was reconstituted using dulbecco’s modified eagle medium (DMEM, Hope Bio-Technology, Qingdao, China) complete medium and cultured in a constant temperature incubator at 37 °C, 5% CO_2_ for five passages. Cells with healthy growth and 90% confluence were digested with 0.25% trypsin-EDTA, then inoculated into six-well plates. After reaching monolayer formation, the culture was transferred to incomplete DMEM without fetal bovine serum and bispecific antibodies for 1 h at 37 °C. The medium was aspirated and cells were washed three times with sterile PBS buffer (pH 7.0, Sangon Biotech, Shanghai, China).

### 2.2. Morphological and Biochemical Analysis

Single colonies of BGI-N8 and BGI-N9, grown on MRS agar plates at 37 °C for 18 h, were selected for Gram staining and endospore staining then examined under an optical microscope (Nikon Ni-u, Nikon Corporation, Tokyo, Japan). Subsequently, the activated bacterial cultures were centrifuged at 10,000 rpm for 10 min at 4 °C. The resulting pellets were washed three times with sterile water and fixed with 2.5% glutaraldehyde for 4 h at 4 °C. Finally, the fixed cells were dehydrated through a graded ethanol series (30%, 50%, 70%, 90%, and 100%) prior to observation and imaging under an electron microscope (Hitachi SU8100, Hitachi Ltd., Tokyo, Japan). The biochemical profiles of BGI-N8 and BGI-N9 were characterized using API 50 CHL test strips (bioMérieux, Marcy l’Etoile, France), according to the manufacturer’s instructions.

### 2.3. Complete Genome Extraction, Sequencing, and Assembly

Strains BGI-N8 and BGI-N9 were cultured in MRS broth at 37 °C. Genomic DNA was extracted from the bacterial cultures using a Rapid Bacterial Genomic DNA Isolation Kit (Sangon Biotech, Shanghai, China) according to the manufacturer’s instructions. For preliminary species identification, the 16S rDNA gene was amplified from the extracted DNA using universal primers 27F (5′-AGAGTTTGATCCTGGCTCAG-3′) and 1492R (5′-GGTTACCTTGTTACGACTT-3′). The PCR amplification was performed under the following conditions: initial denaturation at 94 °C for 5 min; 35 cycles of denaturation at 94 °C for 30 s, annealing at 56 °C for 30 s, and extension at 72 °C for 30 s; followed by a final extension at 72 °C for 10 min. The PCR products were sequenced by Sanger sequencing (BGI write Co., Ltd., Beijing, China) using the 27F and 1492R primers. Final species-level assignment was supported by whole-genome sequencing and ANI analysis. The resulting sequences were analyzed using Sequence Scanner v1.0 and subjected to BLAST analysis using the NCBI BLAST web server (https://blast.ncbi.nlm.nih.gov/Blast.cgi; accessed on 1 July 2026) for species confirmation.

For comprehensive genome analysis, the genomic DNA of both strains was subjected to whole-genome sequencing. Short-read sequencing was conducted on the DNBSEQ-T1 platform (MGI Tech, Shenzhen 518083, China), while long-read sequencing was performed on the CycloneSEQ-WT02 platform (BGI Hangzhou CycloneSEQ Technology Co., Ltd., Hangzhou 310030, China). The raw sequencing data were processed through a quality control pipeline. For short reads, fastp v0.23.4 was used to remove sequences shorter than 90 bp, those containing more than three ambiguous bases, and adapter sequences (AAGTCGGAGGCCAAGCGGTCTTAGGAAGACAA, AAGTCGGATCGTAGCCATGTCGTCGTTCTGTGAGCCAAGGAGTTG), with a quality threshold of Q20 > 97.5%. For long reads, NanoFilt v2.8.0 was applied to filter out reads with a quality score below 10 or shorter than 1000 bp. A complete genome sequence for each strain was subsequently generated by performing a hybrid assembly of the quality-filtered short-read and long-read data using Unicycler v0.4.8. The quality of the assembled genomes was assessed using CheckM2 v1.0.2. Pseudogenes and CRISPR loci were analyzed using Bakta 1.12.0 during genome annotation, and the corresponding feature counts were extracted from the Bakta annotation summary.

### 2.4. Phylogenetic Analysis

To clarify the phylogenetic relationship between the BGI-N8 and BGI-N9 strains, a phylogenetic tree utilizing whole genome sequences was constructed. The genomes of the query strains, along with related strains sourced from NCBI, including 119 strains of *Pediococcus* species from CGR2 were systematically classified using the GTDB-Tk v2.4. tool. The phylogenetic tree was inferred with FastTree v2.1.11 software, while visual presentation and annotation were facilitated by the interactive Tree of Life (iTOL) platform [11].

### 2.5. Genome Annotation

The genomes of BGI-N8 and BGI-N9 were annotated using Prokka v1.14.6, with circular genome maps constructed through the Proksee server (https://proksee.ca/; accessed on 1 July 2026). Gene prediction was further performed using GeneMarkS 4.28. The Average Nucleotide Identity (ANI) was analyzed with FastANI. For functional classification, Clusters of Orthologous Groups (COG) analysis was conducted via the EggNOG database (http://eggnog5.embl.de/; accessed on 1 July 2026), and full genome functional annotation was completed using the Kyoto Encyclopedia of Genes and Genomes (KEGG) Mapper Reconstruct tool (https://www.genome.jp/kegg/mapper/reconstruct.html; accessed on 1 July 2026). Potential genes for carbohydrate-active enzymes (CAZymes) were predicted using the HMMER database in the dbCAN3 server (https://pro.unl.edu/dbCAN2/; accessed on 1 July 2026), with parameters set to an E-value < 0.001 and coverage > 0.35. The prediction of potential bacteriocin-producing gene clusters was conducted using the BAGEL4 database (http://bagel4.molgenrug.nl/; accessed on 1 July 2026) and the bacterial version of the antiSMASH database (https://antismash.secondarymetabolites.org/; accessed on 1 July 2026). Adhesion-related genes were annotated by performing a BLASTP search (parameters: -evalue 1000 -word_size 2 -matrix PAM30 -gapopen 9 -gapextend 1) against a curated set of adhesin sequences from the UniProt database. Key metabolic pathways, such as acetate biosynthesis, were reconstructed by mapping EC numbers from the EggNOG annotations to KEGG pathways.

For safety assessment, virulence factors and antibiotic resistance genes were screened against the Virulence Factor Database (VFDB, http://www.mgc.ac.cn/VFs/; accessed on 1 July 2026) and the Comprehensive Antibiotic Resistance Database (CARD, https://card.mcmaster.ca; accessed on 1 July 2026) using its Resistance Gene Identifier (RGI) tool (version 6.0.5), respectively. For each hit, the RGI detection category, CARD model type, curated bit-score cutoff, best-hit bit-score, percentage identity, reference sequence coverage, drug class, and resistance mechanism were recorded. Perfect and Strict hits were considered high-confidence predictions, whereas Loose hits were retained only for supplementary inspection and were not interpreted as confirmed functional resistance determinants [12]. To evaluate potential transferability, the ±10 kb flanking regions of each putative AMR-related hit were inspected for mobile genetic elements, including transposases, integrases, recombinases, insertion sequences, plasmid-associated genes, and conjugation-related genes, based on Bakta genome annotation and MobileElementFinder analysis.

### 2.6. In Vitro Gastrointestinal Transit Tolerance

Referring to the method of Wang et al. [13], the gastrointestinal tolerance of the strains was evaluated by measuring their survival rates after exposure to simulated stressful conditions, including 0.3% (*w*/*v*) bile salts, artificial intestinal fluid, and artificial gastric fluid (pH 2.0 and 3.0). For bile salt tolerance, bacterial suspensions were mixed with sterile MRS medium containing 0.3% (*w*/*v*) bile salts. The artificial intestinal fluid was prepared using an inorganic salt solution supplemented with NaCl and trypsin at a final activity of 180 U/mL. The artificial gastric fluid was prepared using an inorganic salt solution supplemented with NH_12_CO_3_, NaCI, tryptone, L-cysteine hydro-chloride and pepsin at a final activity of 2500 U/mL. The pH was adjusted to 2.0 or 3.0 using sterile HCl. The inorganic salt mixture contained KCI, KH_2_PO_4_, NaHCO_3_,MgCl_2_(H_2_O)_6_, and CaCl_2_(H_2_O)_2_. They were all filtered by the 0.22 μm filter. The reagents used were purchased from Thermo Fisher Scientific, Waltham, MA, USA and Yuanye Bio-Technology, Shanghai, China.

Overnight cultures of BGI-N8 and BGI-N9 were harvested by centrifugation at 7000 rpm for 5 min at 4 °C and washed twice with sterile PBS. The cell pellets were resuspended in PBS, and the OD_600_ was adjusted to 0.7. The bacterial suspensions were separately mixed with 0.3% bile salt solution, artificial gastric fluid at pH 2.0 or 3.0, and artificial intestinal fluid, followed by incubation at 37 °C for 2 h. Viable counts at 0 and 2 h were determined by plate counting. The survival rate was calculated as follows: Survival rate (%) = N_2_h/N_0_h × 100% where N_0_h and N_2_h represent the viable counts before and after treatment, respectively.

### 2.7. Antibacterial Activity Test

The antibacterial activity of strains BGI-N8 and BGI-N9 against four gut-associated opportunistic pathogens (*Escherichia coli*, *Staphylococcus aureus*, *Pseudomonas aeruginosa*, and *Enterobacter cloacae*) was assessed using a supernatant inhibition assay in 96-well plates [14]. These indicator strains were selected to represent both Gram-negative and Gram-positive intestinal or foodborne opportunistic pathogens.

To be specific, overnight cultured BGI-N8, BGI-N9 and pathogens were centrifuged at 7000 rpm for 5 min at 4 °C, and the rough supernatants and cells of pathogens were collected. Then, the supernatants got filterd through the 0.22 μm filter and each pathogen was adjusted to approximately 0.7 of OD_600_ value. For the inhibition assay, pathogen growth medium was mixed with either sterile water or bacterial culture supernatant at a ratio of 1:1 (*v*/*v*) in 96-well plates. The pathogen suspension was then added to each well at the same inoculum level. The growth control contained pathogen culture medium and sterile water without probiotic culture supernatant, while the blank control contained uninoculated medium. Plates were incubated at 37 °C for 24 h, and absorbance at 600 nm was measured at 0 h and 24 h using a microplate reader.

The inhibition rate was calculated according to the following formula:

Inhibition rate (%) = [1 − (A_T24_ − A_T0_)/(A_N24_ − A_N0_)] × 100%

where A_T24_ and A_T0_ represent the OD_600_ values of the treatment group containing bacterial culture supernatant after 24 h and at 0 h, respectively; A_N24_ and A_N0_ represent the OD_600_ values of the growth control group after 24 h and at 0 h, respectively. Results were expressed as mean ± SD from three technical replicates.

### 2.8. Bacterial Auto-Aggregation Assay

The adhesion capabilities of the two strains were evaluated through cell auto-aggregation experiments [15]. Briefly, BGI-N8 and BGI-N9 were activated overnight in MRS broth at 37 °C. The bacterial cells were harvested by centrifugation (6000× *g*, 10 min, 4 °C), washed twice with sterile phosphate-buffered saline (PBS, pH 7.2), and resuspended in the same buffer to an optical density (OD) of approximately 1.0 at 600 nm (initial OD, designated as A_0_). Subsequently, 4 mL of the bacterial suspension was transferred into a sterile test tube and allowed to stand undisturbed at room temperature for 24 h. The OD_600_ of the supernatant was measured at 0, 6, 12, and 24 h (designated as A_t_). The auto-aggregation rate was calculated using the following formula: Auto-aggregation rate (%) = [1 − (A_t_/A_0_)] × 100%.

### 2.9. Cell Adhesion Assay

The further adhesive abilities of BGI-N8 and BGI-N9 to HT-29 human colon carcinoma cells were assessed as previously described with modifications [16]. HT-29 cells were seeded into six-well plates at approximately 1 × 10^6^ cells per well and cultured until a confluent monolayer was formed. Before the adhesion assay, the cell monolayers were washed with sterile PBS and maintained in incomplete DMEM. Bacterial suspensions of BGI-N8 and BGI-N9 were prepared at a concentration of 1 × 10^8^ CFU/mL Based on the bacterial inoculum and HT-29 cell number used, the approximate multiplicity of infection was 100:1. For each strain, 500 μL of the bacterial suspension was mixed with 500 μL of incomplete DMEM and added to the six-well plates containing HT-29 cell monolayers, followed by a 1.5-h incubation at 37 °C. Blank control and positive control groups were established by replacing the test bacterial suspensions with sterile PBS and an equivalent concentration of *L. paracasei* ATCC 334 suspension, respectively [17]. Post-incubation, the cells were washed five times with sterile PBS to remove non-adherent bacteria. Subsequently, the cells were fixed with 10% formaldehyde for 2 h at room temperature. After fixation and drying, Gram staining was performed. Twenty random microscopic fields were examined, and bacterial attachment index was calculated as: Adhesion index = Number of bacteria/Number of cells.

### 2.10. Determination of the Ability of the Strain to Inhibit α-Glucosidase

The α-glucosidase inhibitory activity of BGI-N8 and BGI-N9 was assessed as follows. To be specific, Overnight cultures were adjusted to an OD_600_ value of 1.0 using sterile PBS, serving as the bacterial suspensions for subsequent assays. For each reaction, 50 μL of 0.1 mol/L PBS (pH 6.8), 50 μL of 20 mmol/L p-nitrophenyl-α-D-glucopyranoside (pNPG), and 25 μL of bacterial suspension were mixed in a 96-well plate and pre-incubated at 37 °C for 10 min. For the control groups, the bacterial suspension was replaced with sterile PBS containing acarbose at final concentrations of 20 μg/mL, 40 μg/mL and 100 μg/mL, respectively. Subsequently 30 μL of α-glucosidase solution (20 U/mL) was added to initiate the reaction. After 20 min of incubation, the reaction was terminated by adding 50 μL of Na_2_CO_3_ solution (1 mol/L). The absorbance was measured at 405 nm. The inhibitory rate was calculated using the following formula:

Inhibition rate (%) = [1 − (A − B)/(C − D)] × 100%

Note: A is the absorbance of the experimental group (with bacterial suspension and α-glucosidase). B is the absorbance of the bacterial suspension control (with bacterial suspension, without α-glucosidase). C is the absorbance of the positive control (with α-glucosidase, without bacterial suspension). D is the absorbance of the blank control (without bacterial suspension and α-glucosidase).

### 2.11. Determination of Cholesterol-Lowering Ability of Strains

The ability of BGI-N8 and BGI-N9 to reduce cholesterol levels was evaluated in MRS-CHOL medium. This medium was prepared by supplementing MRS broth with sodium thioglycolate (2 g/L), bile salts (0.3%, Oxgall), and cholesterol (120 μg/mL). Activated cultures of BGI-N8 or BGI-N9 were inoculated at 10% (*v*/*v*) into 2 mL of the MRS-CHOL medium to constitute the experimental groups, while an uninoculated medium served as the control. All samples were incubated at 37 °C for 48 h. Then, they were centrifuged at 4000× *g* for 15 min. The cholesterol content in the supernatant was measured using a total cholesterol assay kit (Sangon Biotech). The cholesterol-lowering capacity of each strain was expressed as the percentage reduction in cholesterol relative to the control.

### 2.12. Statistical Analysis

All in vitro experiments were performed in technical triplicate, and the data were expressed as mean ± standard deviation (SD). SPSS Statistics 26.0 (IBM, Chicago, IL, USA) and GraphPad Prism v9.0.0.121 (San Diego, CA, USA) were used for statistical analysis and plotting. For comparisons involving more than two groups, one-way analysis of variance followed by Tukey’s test post hoc was used. For pairwise comparisons between two groups, an unpaired Student’s *t*-test was used when appropriate. Normality and homogeneity of variance were evaluated using the Shapiro–Wilk test and Levene’s test, respectively. A value of *p* < 0.05 was considered statistically significant.

## 3. Results

### 3.1. Characteristics of BGI-N8 and BGI-N9

**Figure 1:**
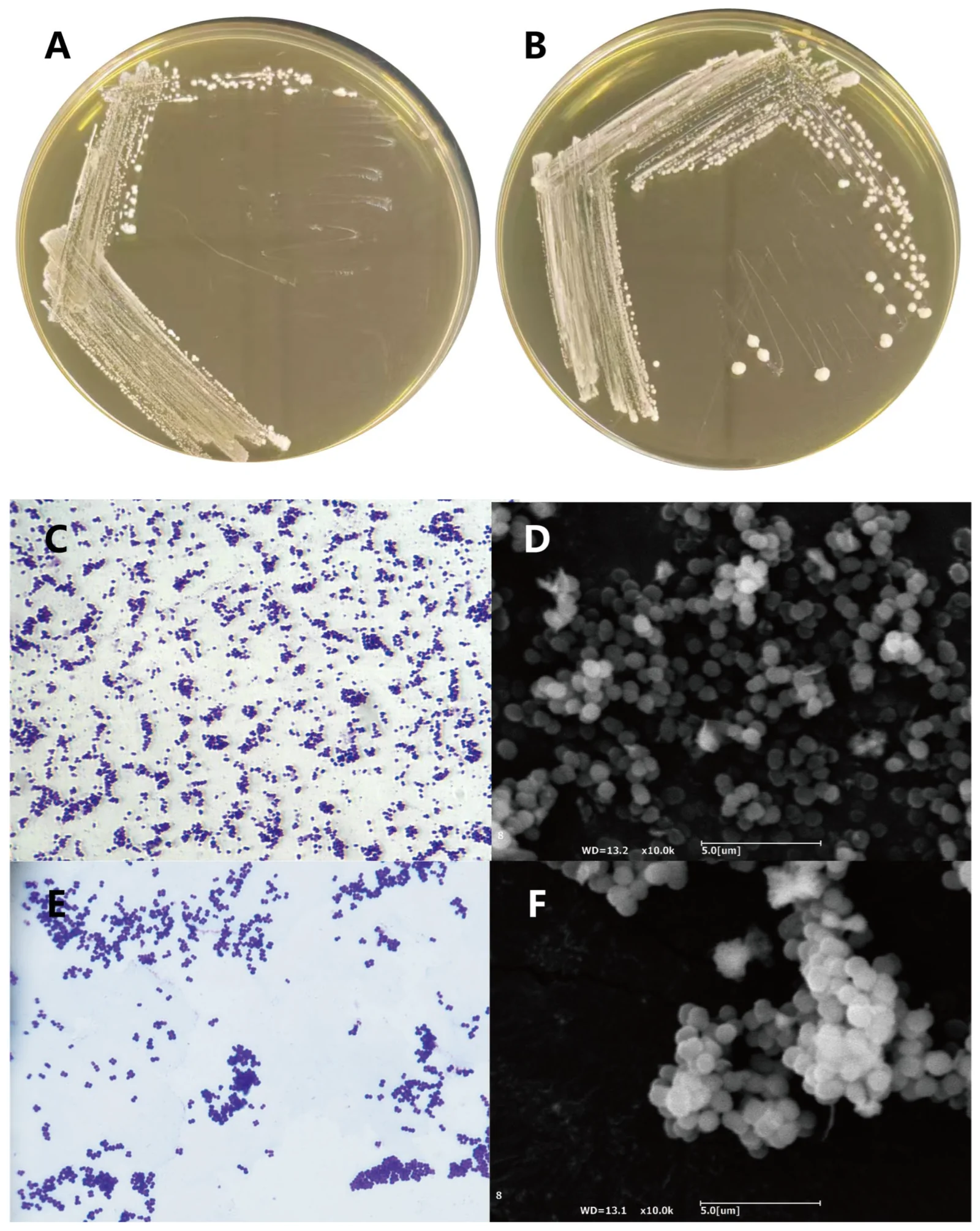
BGI-N8 and BGI-N9 morphological characteristics. Colony morphology of BGI-N8 (**A**) and BGI-N9 (**B**). Gram staining of BGI-N8 (**C**) and BGI-N9 (**E**) observed under a optical microscope (1000×). Scanning electron microscopy (SEM) images of BGI-N8 (**D**) and BGI-N9 (**F**) (10,000×).

After 18 h of incubation on MRS agar at 37 °C, both strains formed moist, smooth-surfaced, opaque colonies with distinct, flat edges. BGI-N8 colonies appeared milky white with smaller diameters of 0.8–1.4 mm (Figure 1A). In contrast, BGI-N9 colonies were milky white with a slight yellow tint, 2–3 mm in diameter, and more raised (Figure 1B). Gram staining and endospore staining confirmed both strains are Gram-positive *cocci* and non-spore-forming. BGI-N8 cells were mostly spherical or ovoid, arranged in pairs or small dispersed aggregates with no chains (Figure 1C). BGI-N9 cells were spherical, arranged in pairs, tetrads, or irregular clusters, also with no chains (Figure 1E). Electron microscopy confirmed both strains are non-flagellated (Figure 1D–F).

### 3.2. Phylogenetic Relationships of BGI-N8 and BGI-N9

**Figure 2:**
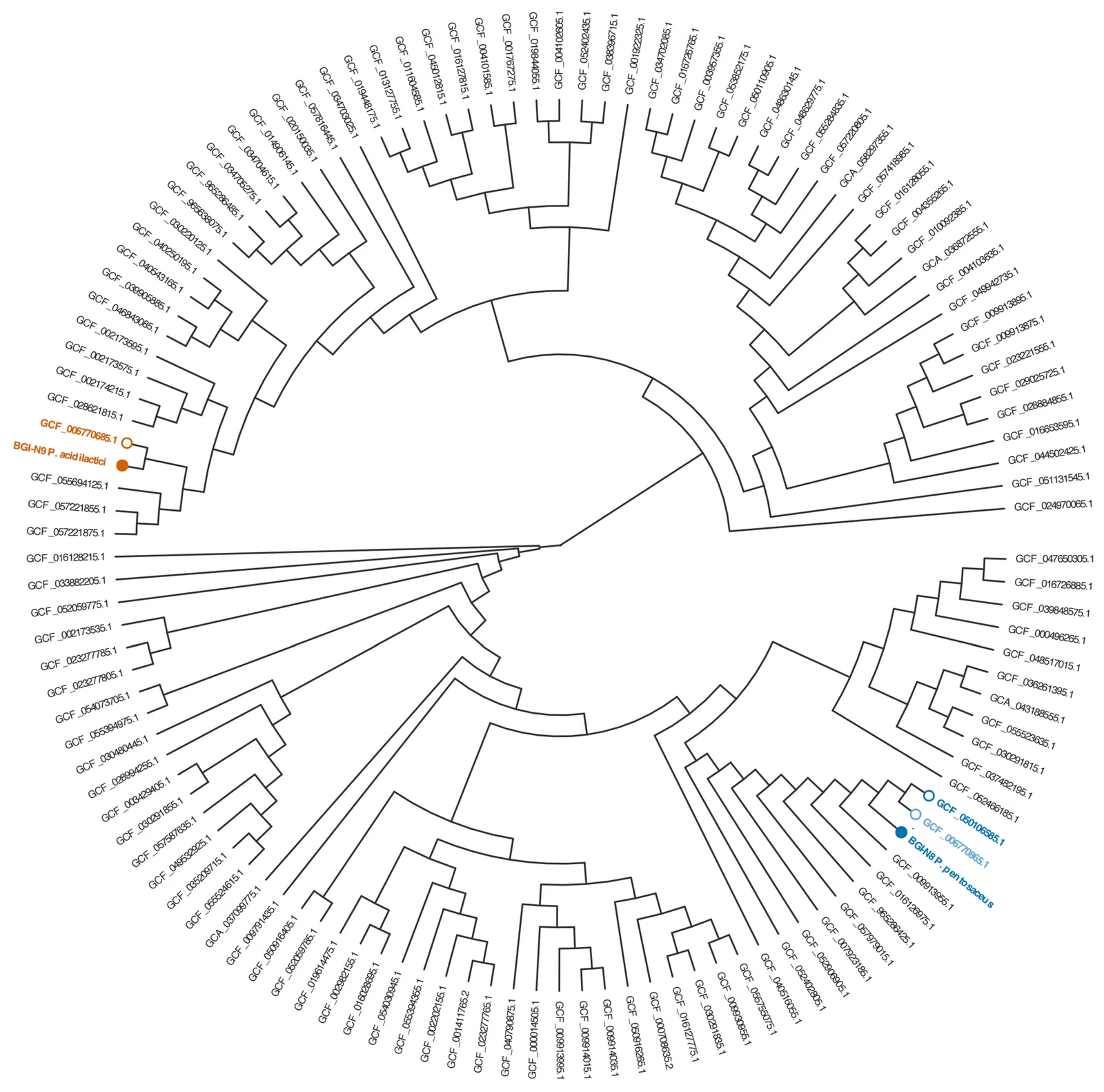
Phylogenetic analysis and taxonomic identification of BGI-N8 and BGI-N9 (highlighted in blue and orange). Colors are used solely for visual highlighting and have no additional biological meaning.

The phylogenetic relationships and precise taxonomic positions of strains BGI-N8 and BGI-N9 were determined through whole-genome sequencing. BGI-N9 showed a certain genetic distance to the outgroup BGI-N8, consistent with evolutionary divergence within the genus (Figure 2). The dendrogram was constructed based on pairwise ANI distances calculated by FastANI. BGI-N8 clustered with the reference genome of *Pediococcus pentosaceus* while BGI-N9 clustered with the reference genome of *Pediococcus acidilactici*, supporting their species-level taxonomic assignments. The ANI values between BGI-N8 and its closest *P. pentosaceus* reference genome and between BGI-N9 and its closest *P. acidilactici* reference genome were >99.99% and >99.99%, respectively, both exceeding the commonly used 95–96% ANI threshold for prokaryotic species delineation [18]. In contrast, the ANI value between BGI-N8 and BGI-N9 was below this threshold, supporting their assignment to two distinct *Pediococcus* species.

### 3.3. Complete Genome Characteristics and Gene Annotations of BGI-N8 and BGI-N9

**Figure 3:**
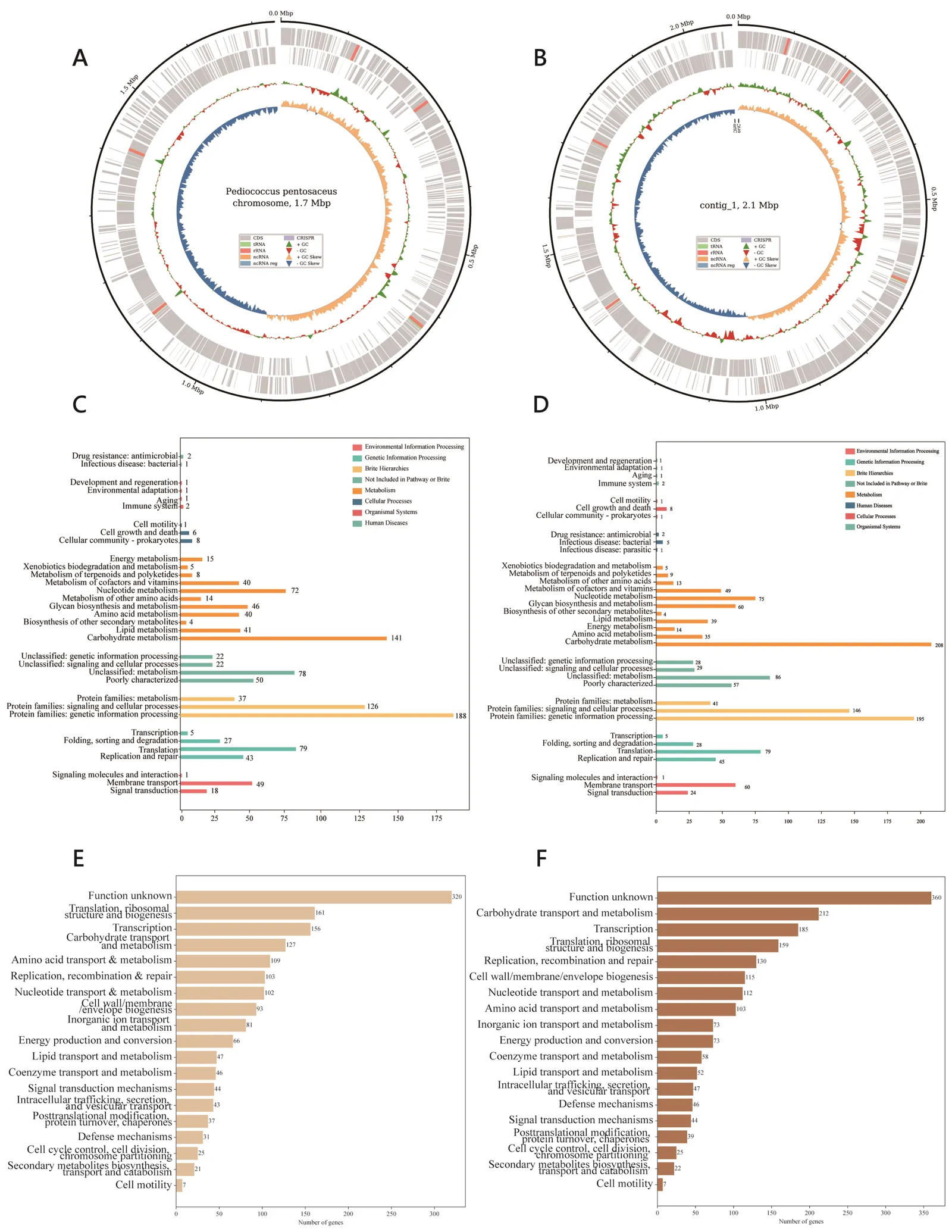
Genomic features and functional annotation of BGI-N8 and BGI-N9. Circular genome maps of BGI-N8 (**A**) and BGI-N9 (**B**). From outer to inner circles: forward-strand CDS, reverse-strand CDS, RNA genes, GC content, and GC skew. Functional classification of predicted genes based on the KEGG database for BGI-N8 (**C**) and BGI-N9 (**D**). Functional classification based on the COG database for BGI-N8 (**E**) and BGI-N9 (**F**).

Genomic features of BGI-N8 and BGI-N9 are summarized in Figure 3A,B. GeneMarks analysis revealed that the total genome length of the BGI-N8 strain was assembled as a single 1,733,038 bp sequence with an N50 of 1,733,038 bp, 100.0% completeness, 0.08% contamination. Its GC content is 37%, with 1698 coding sequences (CDS) identified. After genome annotation using the Prokka software, it was found that this strain harbors 15 rRNA genes, 56 tRNA genes, and 1 tmRNA gene. BGI-N9 was assembled as a single 2,085,606 bp contig with an N50 of 2,085,606 bp, 100.0% completeness, and 0.48% contamination. A total of 1956 CDS, 15 rRNA genes, 57 tRNA genes, and 1 tmRNA gene were identified through prediction. Both genomes have GC contents within the typical range for lactic acid bacteria (33–55%), and no plasmids were detected in either strain. Annotation analysis by Bakta revealed that no predicted CRISPR loci either pseudogene were identified in either BGI-N9 or BGI-N8; additional genomic details are provided in Table 1.

According to the KEGG metabolic pathway annotation, analysis revealed that the coding genes of BGI-N8 were categorized into 34 functional groups, while those of BGI-N9 were divided into 35 functional groups, as shown in the Figure 3C,D. Carbohydrate metabolism genes were the most abundant in both strains, followed by genes involved in genetic information processing, amino acid metabolism, and vitamin metabolism. COG analysis showed the largest category was “functionally unknown” in both strains. Carbohydrate transport and metabolism was the most common core functional category, with additional genes involved in transcription, translation, and ribosomal structure (Figure 3E,F).

### 3.4. Utilization of Carbohydrates

**Figure 4:**
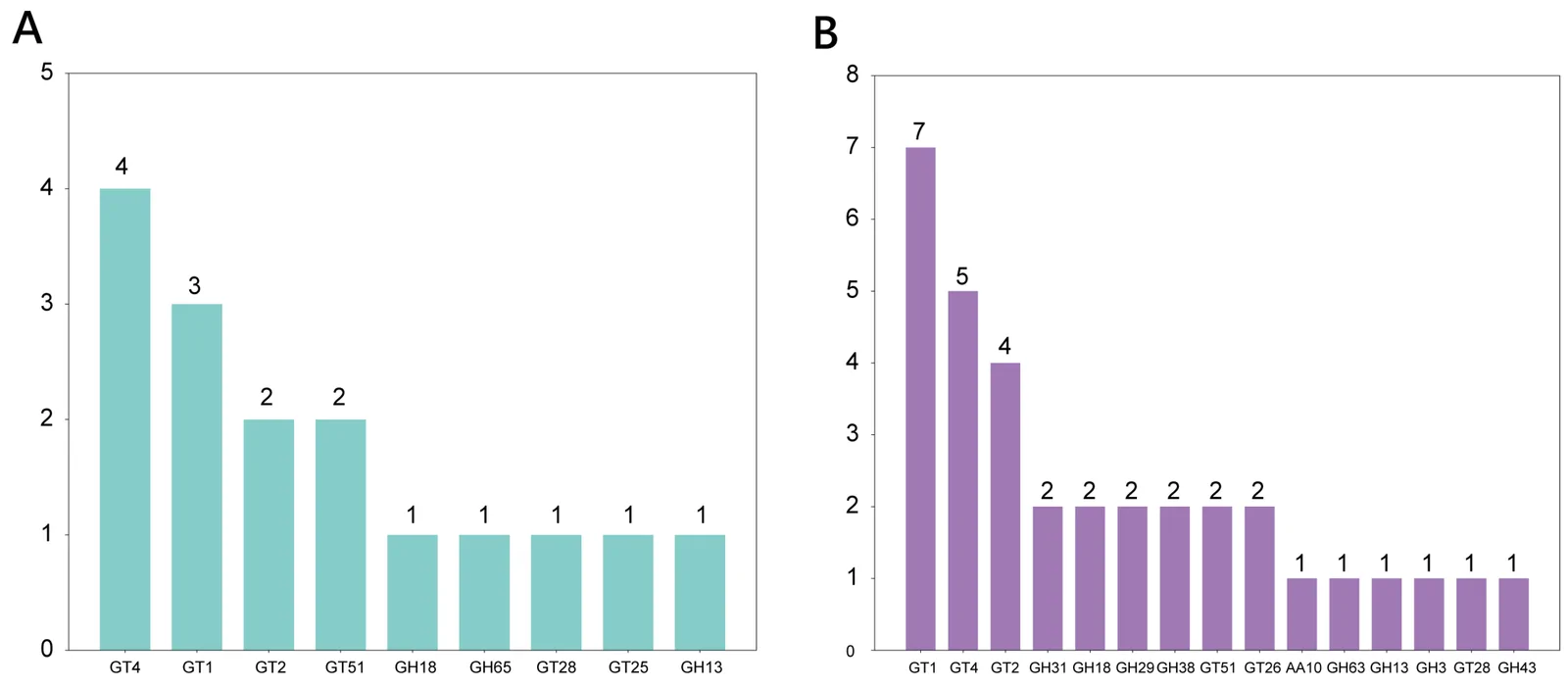
Genomic analysis of carbohydrate-active enzymes (CAZy) in BGI-N8 (**A**) and BGI-N9 (**B**).

Carbohydrate-active enzymes (CAZymes) mediate carbohydrate assembly, degradation, and modification. CAZyme profiles for BGI-N8 and BGI-N9 are shown in Figure 4A,B. BGI-N8 encodes multiple CAZymes, with glycosyltransferase (GT) families GT4 and GT1 most abundant, followed by GT2, GT51, GT28, and GT26. Glycoside hydrolase (GH) families GH18, GH65, and GH13 each have one gene. BGI-N9 encodes 34 CAZymes, including 21 GT family genes (GT1, GT4, and GT2 most abundant; GT51, GT26, and GT28 less abundant). BGI-N9 also has 12 GH family genes (GH31, GH18, GH29, and GH38 with two genes each; GH63, GH13, GH3, and GH43 with one gene each) and one Auxiliary Activities family AA10 gene.

Both strains encode multiple genes for galactose, glucose, and fructose metabolism (Table S1). The genes *galK*, *pgm*, *galR*, *galE*, *galT*, *glcK*, *glcU*, and *fruA* are the common carbon source metabolizing genes of the two strains, while *fruR* is a unique fructose metabolizing gene of the BGI-N9 strain. These genes encode different enzymes that participate in the metabolic processes of glucose, galactose, and fructose. The carbon source utilization test shown in Table S2 echoed the above analysis. Both strains utilized L-arabinose, ribose, galactose, fructose, dextrin, salicin, and trehalose. BGI-N9 additionally utilized D-xylose, while BGI-N8 utilized lactose, arbutin, and maltose. Neither strain utilized adonitol, sorbitol, or mannitol.

### 3.5. Genome Safety Analysis

The genomic safety of both BGI-N8 and BGI-N9 was assessed by screening their genomes against the Virulence Factor Database (VFDB). Employing stringent criteria (sequence similarity > 90% and coverage > 80%) to identify high-risk and dedicated virulence determinants, no canonical virulence factors (e.g., toxins, invasins, or secretion system effectors) were detected in either genome. Under relaxed homology thresholds (similarity > 50%), a set of genes associated with basic cellular functions exhibited matches to entries in the VFDB (Table S3). This conserved set includes genes involved in stress survival (e.g., *clpC*, *clpE*, *clpP*), cell surface biosynthesis/ immunomodulation (e.g., *hasC*, *cpsA*/*uppS*), and adhesion(e.g., *groEL*, *tufA*). BGI-N8 and BGI-N9 shared a highly congruent profile of these genes. Nine additional genes were uniquely predicted in BGI-N9 under the relaxed threshold, though their predicted functions also fall within general metabolic or stress-response categories. Genome resistance gene analysis (as shown in Table S4) identified no Perfect AMR hits in either genome. Only several Strict, low-identity AMR-related homologous hits were predicted, including *vanT* in BGI-N8 and *sdrM*, *vanT*, and *qacG* in BGI-N9. Because these hits showed relatively low sequence identity and were not supported by evidence of adjacent mobile genetic elements, they were not interpreted as confirmed transferable resistance genes.

### 3.6. Analysis of Digestive Tract Environmental Tolerance

**Figure 5:**
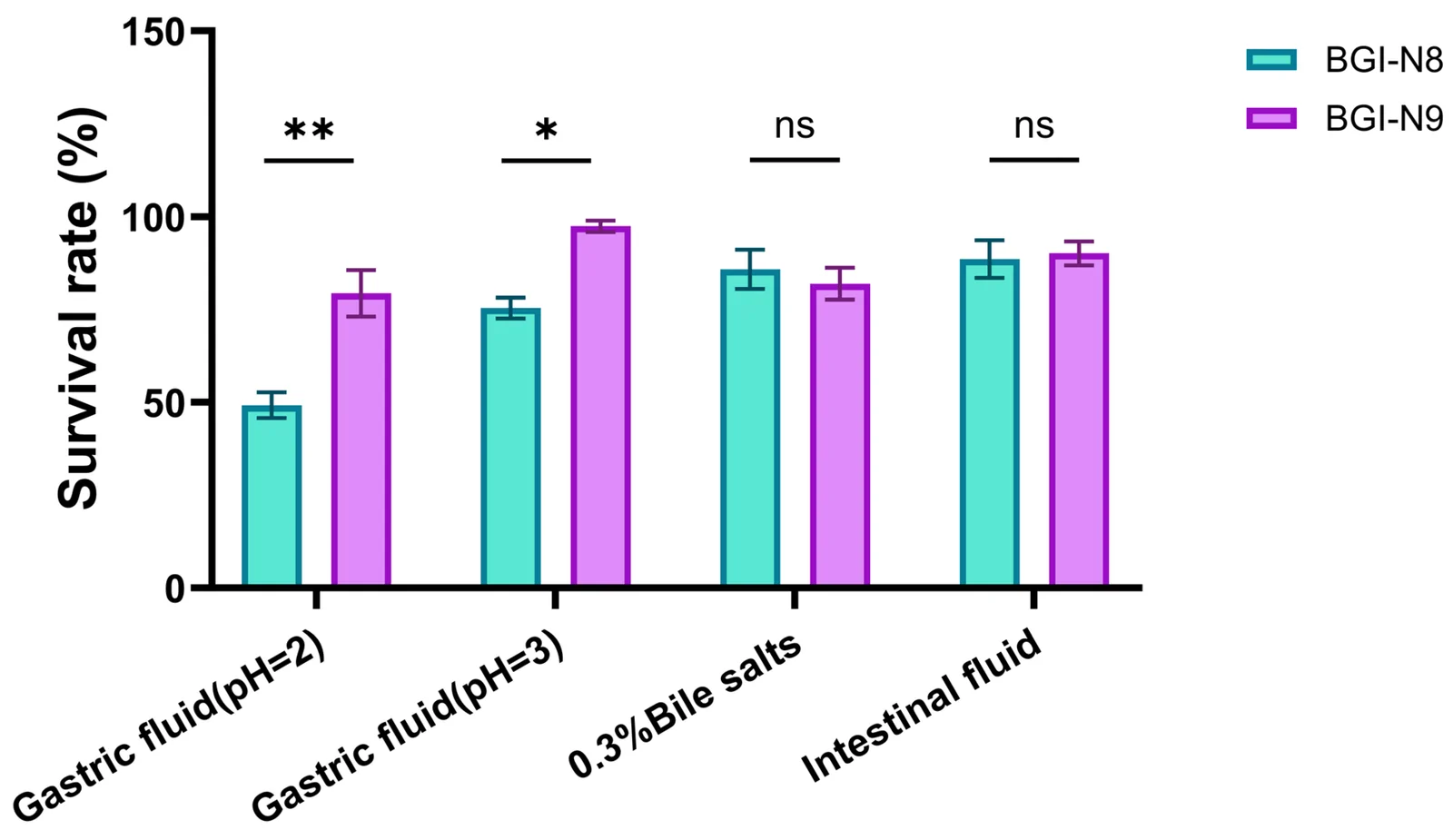
Survival rates of BGI-N8 and BGI-N9 after exposure to simulated gastric fluid (pH 2.0, 3.0), intestinal fluid, and 0.3% bile salts. Values are mean ± SD (*n* = 3). *—0.01 < *p* < 0.05, **—*p* < 0.01, ns is not significant.

Gastrointestinal tolerance was evaluated through genomic analysis and in vitro assays. Genomic annotation identified complementary sets of genes for acid tolerance, bile resistance, and gut persistence in the two strains (Table S5). BGI-N9 has a more comprehensive acid resistance system, including a complete dltA-D operon, a full set of F0F1-ATPase genes (*atpB-atpH*) and comprehensive stress response systems (e.g., *hrcA-dnaK-grpE* chaperones, clpproteases). In contrast, BGI-N8 primarily relies on the F0F1-ATPase system and a few general stress proteins (e.g., *asp2*) for acid adaptation. For bile salt resistance, BGI-N8 exhibited a broader repertoire of genes potentially involved in bile stress mitigation. BGI-N9 encoded key bile resistance elements such as gene cfa and redox homeostasis genes (*katA*, *tpx*, *msrA/B*, *trxA/B*), but lacked the extensive *opp* system and ribosomal protein enrichment seen in BGI-N8. Both strains harbored genes that may facilitate initial gut colonization and persistence. Shared features include a cellobiose PTS system (*celC*) for carbohydrate utilization, and sortase (*srtA*). In vitro results confirmed these genomic predictions (Figure 5). Both strains exhibited comparable gastrointestinal tolerance abilities, with survival rates exceeding 81% in 0.3% bile salt and simulated intestinal fluid. Notably, BGI-N9 displayed exceptional survival in low-pH conditions. Its survival rates at pH 2.0 and pH 3.0 (79.3% and 97.4%, respectively) were significantly greater than those of BGI-N8 (49.2% and 75.4%; *p* < 0.05).

### 3.7. Antibacterial Ability Analysis

**Figure 6:**
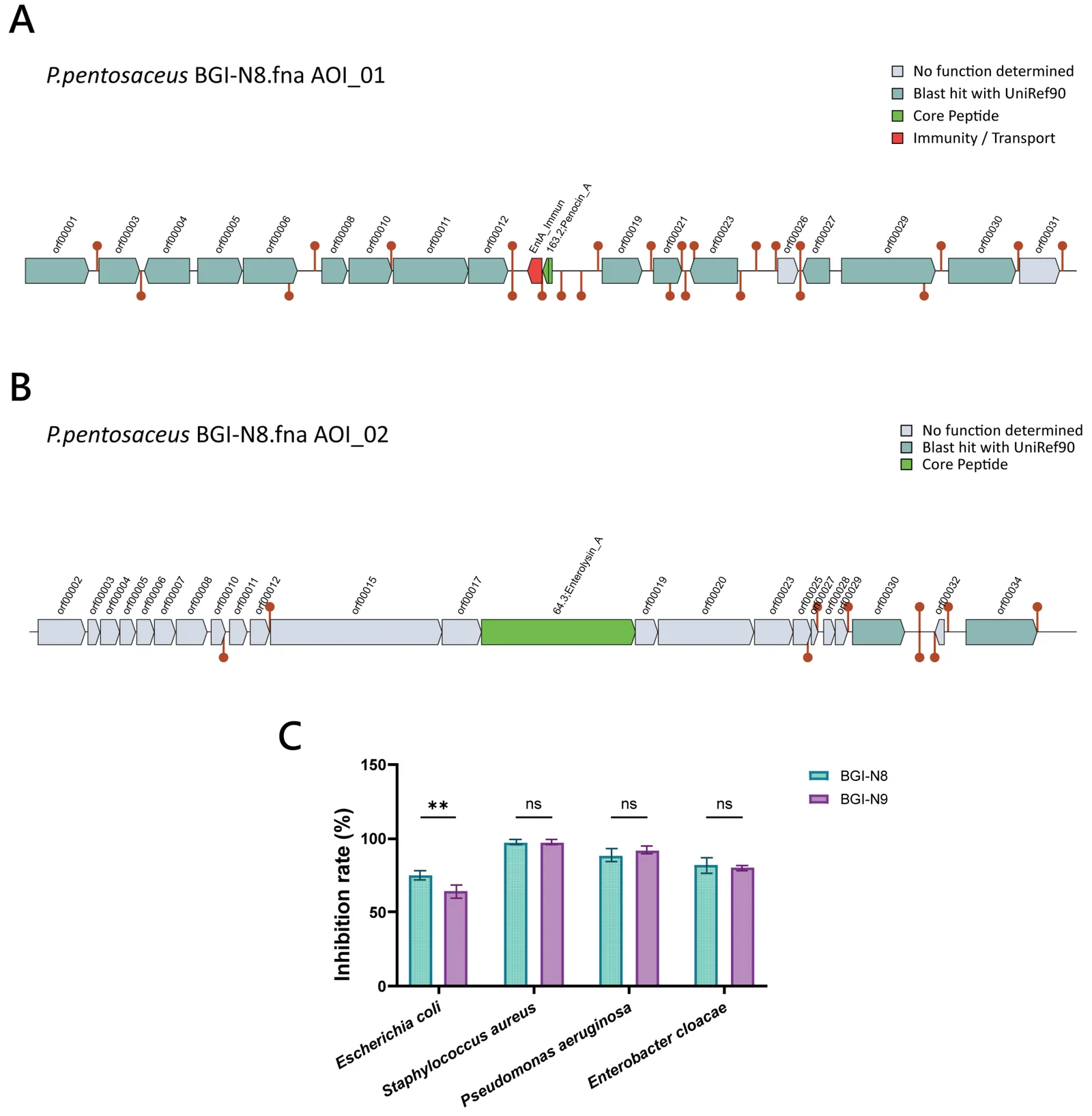
Antibacterial activity analysis of BGI-N8 and BGI-N9. (**A**,**B**) Genetic organization of two putative bacteriocin gene clusters (AOI_01 and AOI_02) identified in the genome of *P. pentosaceus* BGI-N8. Arrows represent open reading frames (ORFs). (**C**) Antibacterial activity of BGI-N8 and BGI-N9 against selected gut pathogens. Data are presented as mean ± SD (*n* = 3). **—*p* < 0.01, ns is not significant.

Analysis of biosynthetic gene clusters (BGCs) using antiSMASH suggested distinct potentials for secondary metabolite production in BGI-N8 and BGI-N9. While both strains encode a polyisoprene synthase (in ctg1_715 of N8 and ctg1_798 of BGI-N9), a key divergence was observed in bacteriocin production. A dedicated gene cluster for class II bacteriocin biosynthesis was identified in the BGI-N8 genome (AOI_01 and AOI_02, Figure 6A,B). Further analysis with BAGEL4 confirmed the cluster’s potential to encode two antibacterial peptides: *Penocin A* and *Enterolysin A*. The annotation results suggest a complete system for bacteriocin maturation and secretion (Figure 6A,B). In contrast, no homologous bacteriocin gene clusters were identified in the BGI-N9 genome, and BAGEL4 analysis did not predict any bacteriocin-coding sequences. in vitro antibacterial assays against common gut pathogens (*E. coli*, *S. aureus*, *P. aeruginosa*, and *E. cloacae*) demonstrated that both strains exhibited substantial inhibitory activity, with inhibition rates ranging from 88.6% to 97.6% (Figure 6C). BGI-N8 showed a significantly stronger inhibitory effect against *E. coli* and *E. cloacae* compared to BGI-N9 (*p* < 0.01, Figure 6C). No significant difference was observed in the inhibition of *S. aureus* and *P. aeruginosa* between the two strains.

### 3.8. Adhesion Capacity Analysis

**Figure 7:**
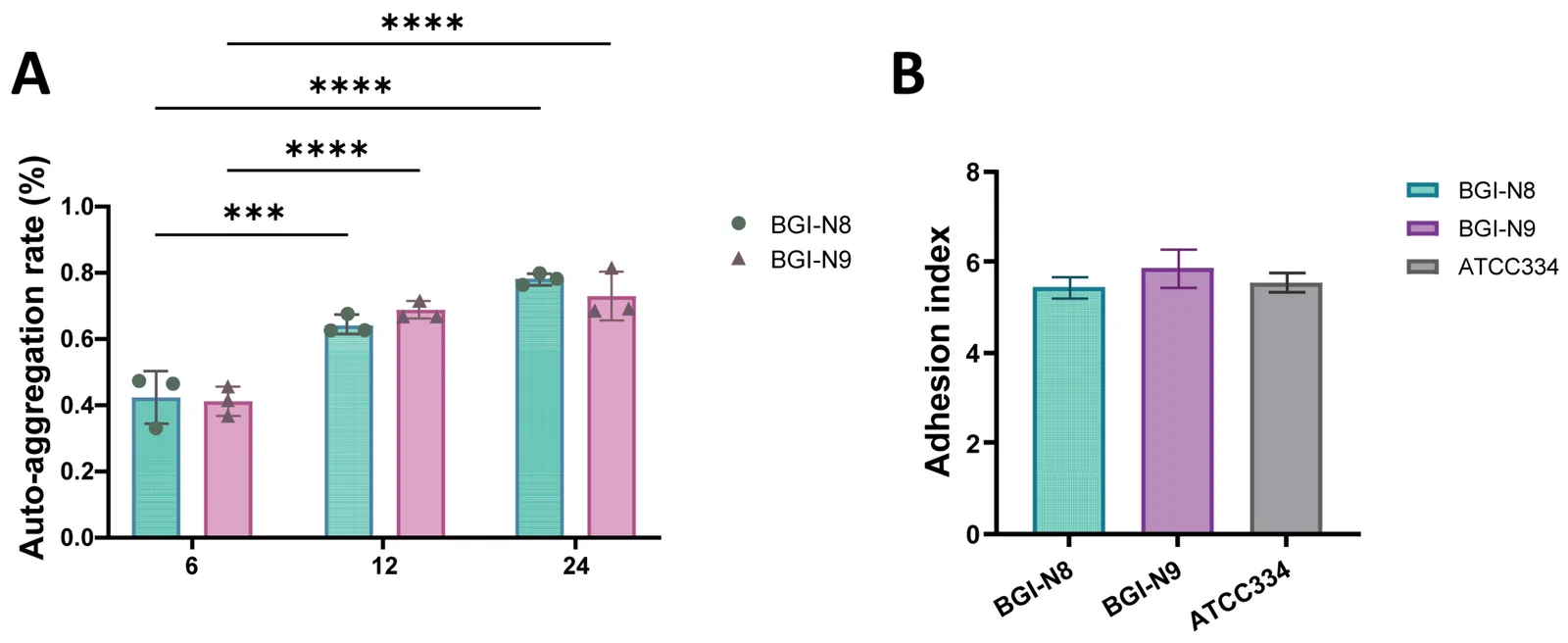
(**A**) Auto-aggregation abilities of BGI-N8 and BGI-N9. (**B**) Comparison of the adhesion capabilities of BGI-N8, BGI-N9, and ATCC 334 to HT-29 Cells. Values are presented as mean ± SD. ***—0.0001 < *p* < 0.001, ****—*p* < 0.0001.

Genomic analysis via BLASTp and Uniprot annotation identified a distinct repertoire of adhesion-associated genes—specifically, 31 genes in BGI-N8 and 35 in BGI-N9 (Table S6)—highlighting the adhesion potential of both strains. To validate these genomic predictions, auto-aggregation assays indicated that strains BGI-N8 and BGI-N9 both achieved aggregation rates above 70% within 24 h (Figure 7A). The auto-aggregation capability of BGI-N8 was more prominent in the later stage (24 h), while BGI-N9 exhibited a more stable trend by the mid-stage (12 h). Furthermore, adhesion assays were performed using the HT-29 human colon cancer cell line. The results showed that BGI-N8 and BGI-N9 exhibited adhesion indices of 5.4 and 5.9, respectively (Figure 7B). These values were statistically comparable to the commercial probiotic *L. paracasei* ATCC 334. (*p* > 0.05).

### 3.9. Evaluation of Hypoglycemic and Hypolipidemic Potential

**Figure 8:**
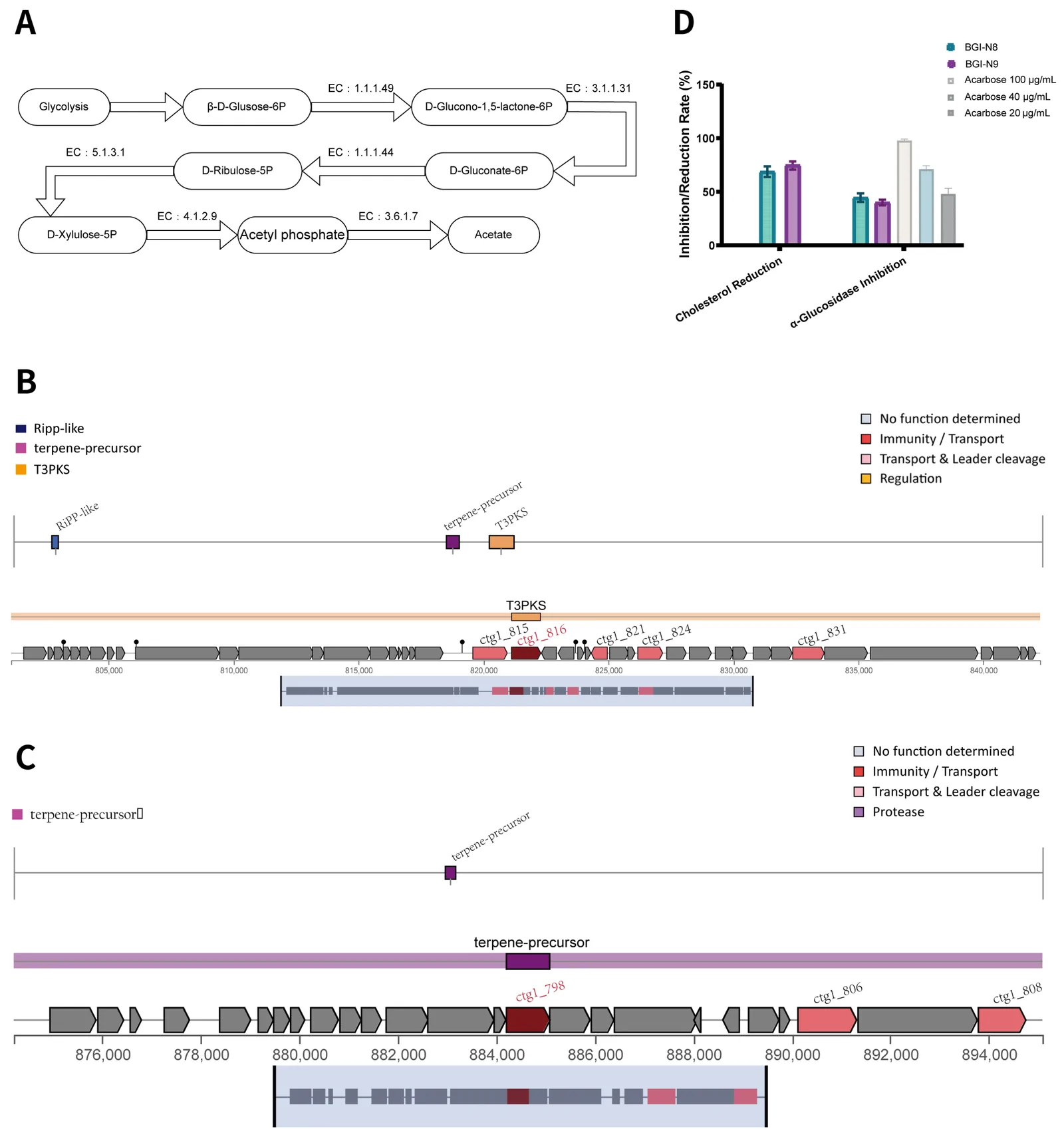
(**A**) The genomic annotation results of BGI-N8 and BGI-N9 in the metabolic-related pathways. Comparative analysis of biosynthetic gene clusters (BGCs) in BGI-N8 (**B**) and BGI-N9 (**C**) using antiSMASH. (**D**) Evaluation of α-glucosidase activity inhibition and cholesterol-lowering potential of BGI-N8, BGI-N9 and acarbose at different concentrations.

Genomic annotation revealed that both BGI-N8 and BGI-N9 possess a rich genetic repertoire associated with pathways for carbohydrate metabolism and cholesterol homeostasis, providing a potential molecular basis for their hypothesized functions. Key annotations include genes for the acetate generation pathway (*zwf*, *pgl*, *gnd*, *rpe*, *xfp*, *acyP*), which ultimately produces acetic acid, a short-chain fatty acid known to modulate host metabolism (Figure 8A). Other related genes are shown in Table S7, specifically the bile salt hydrolase gene (*bsh*) was identified in both strains, which is commonly related to deconjugating bile acids and is considered a key mechanism for promoting cholesterol degradation. Furthermore, the divergent metabolic potentials of BGI-N8 and BGI-N9 were further elucidated through the analysis of biosynthetic gene clusters (BGCs) using antiSMASH. BGI-N8 exhibited a more complex biosynthetic repertoire with three distinct regions, including a *RiPP*-like cluster (Region 1), a terpene-precursor cluster (Region 2), and a unique Type III Polyketide Synthase (T3PKS)-like cluster (Region 3) (Figure 8B). Notably, deeper genomic mining within specific Region 3 of BGI-N8 identified ctg1_816, which encodes a hydroxymethylglutaryl-CoA (HMG-CoA) synthase, a pivotal enzyme in the mevalonate pathway for synthesizing bioactive terpenoids. In contrast, BGI-N9 harbors a streamlined secondary metabolism with only a single terpene-precursor region detected (Figure 8C). The results of the in vitro experiments are shown in Figure 8D. BGI-N8 and BGI-N9 showed α-glucosidase inhibitory activity, with inhibition rates of 44.53% for BGI-N8 and 39.94% for BGI-N9 (*p* > 0.05). Both strains showed comparable cholesterol-removal capacities under the tested in vitro conditions, with removal rates of 68.76% and 74.49%, respectively (*p* > 0.05).

## 4. Discussion

This study used an integrated genomic-phenotypic approach to characterize the probiotic potential of *Pediococcus pentosaceus* BGI-N8 and *Pediococcus acidilactici* BGI-N9. Genomic and phenotypic analyses collectively provided preliminary genomic safety evidence that both isolates, plasmid-free lactic acid bacteria with broad carbon fermentation capabilities, substantiating their potential application as functional ingredients. Both strains meet core probiotic criteria and show distinct advantages in glucose and lipid metabolism regulation.

Safety and genetic stability are essential for probiotic use. Both strains are plasmid-free, which reduces the risk of horizontal gene transfer which may be a major concern for antibiotic resistance dissemination [8,19]. Applying stringent criteria (sequence similarity > 90% and coverage > 80%) to identify high-risk and specific virulence determinants, no canonical virulence factors such as toxins, invasins, or secretion system effectors were identified in either genome. However, relaxing homology thresholds (similarity > 50%) revealed a set of genes linked to fundamental cellular processes that matched entries within the VFDB. For instance, the detected virulence factors (e.g., *clpP*, *groEL*) were detected. However, these genes are mainly associated with stress response and adhesion mechanisms, rather than direct pathogenicity [20,21]. Antimicrobial resistance screening also required cautious interpretation. CARD-RGI analysis identified no perfect antimicrobial resistance hits in either genome. The predicted AMR-related hits, including *vanT* gene in BGI-N8 and *sdrM*, *vanT* and *qacG* in BGI-N9, were classified as low-identity homologous hits rather than confirmed functional resistance genes. Though *vanT* has been associated with putative intrinsic vancomycin resistance [22], the detection of a partial or low-similarity vanT-like hit, even together with other low-identity AMR-related homologous hits such as *sdrM*, does not indicate the presence of a complete or transferable resistance cluster [23]. The detection of a partial or low-similarity *vanT*-like hit alone does not indicate the presence of a complete transferable resistance cluster. Moreover, previous studies indicate that intrinsic vancomycin resistance is a common, non-transferable trait in the genus *Pediococcus* and *Lactobacillus*, often related to cell wall synthesis rather than mobile resistance elements [24]. Although genome-based screening did not identify high-confidence transferable antimicrobial resistance determinants or canonical virulence factors, these findings should be interpreted as a preliminary genomic safety assessment rather than definitive evidence of probiotic safety. Standardized phenotypic antimicrobial susceptibility testing, such as MIC determination or disk-diffusion assays, was not completed in the present study and remains essential for validating the antibiotic susceptibility profiles of BGI-N8 and BGI-N9. In addition, hemolysis testing, biogenic amine production, D-lactate production, mucin degradation, cytotoxicity and other host–cell safety evaluations are still required to further confirm their safety profiles and probiotic application.

Gastrointestinal stress tolerance is an important screening criterion for probiotic candidates. Both BGI-N8 and BGI-N9 exhibited favorable in vitro tolerance to simulated gastrointestinal conditions, employing distinct strengths in acid and bile resistance that may correlate to their genomic profiles. BGI-N9 showed relatively higher acid tolerance under the tested conditions. Genomic annotation provided several possible explanations for these phenotypic differences. For example, BGI-N9 harbored a complete *dltA-D* operon, which mediates the D-alanylation of teichoic acids to effectively repel protons [25,26]. This mechanism may operate in synergy with a complete F0F1-ATPase proton extrusion cluster [27] and comprehensive stress response machineries [28]. BGI-N8 lacks an intact *dlt* operon and may rely primarily on the F0F1-ATPase system for acid tolerance. The two strains use complementary bile resistance strategies. BGI-N8 has an extensive *opp* amino acid transport system for bile salt sensing and efflux [29], plus ribosomal protein genes for compensatory protein synthesis [28]. In contrast, the *cfa* genes (cyclopropane fatty acid synthase [30] and redox homeostasis factors detected in BGI-N9 probably help to counteract bile-induced oxidative damage [31]. However, the direct contribution of these genes to the observed acid-tolerance phenotype was not experimentally verified.

The gut microbiota maintains gut homeostasis, and pathogen overgrowth disrupts this balance by increasing pathogen-associated inflammatory stimuli, including lipopolysaccharide (LPS) [32]. Systemic LPS has been associated with chronic low-grade inflammation, which accelerates metabolic syndrome and related metabolic disorders [33,34]. Therefore, antagonistic activity against pathogenic bacteria is not only a desirable probiotic candidate trait but may also be relevant to microbiota-oriented strategies for metabolic health support. In the present study, crude culture supernatants from both BGI-N8 and BGI-N9 inhibited the growth of the four tested gut-associated indicator pathogens, indicating a preliminary in vitro antimicrobial phenotype. BGI-N8 shows stronger activity against *E. coli* and *E. cloacae* than BGI-N9 (*p* < 0.01), suggesting strain-dependent differences in extracellular inhibitory activity. Genome analysis identified bacteriocin-related genes, including *Penocin A* and *Enterolysin A*, in BGI-N8, whereas such bacteriocin-related genes were not detected in BGI-N9. Previous studies have reported that these *Pediococcus*-derived bacteriocins exert targeted antagonistic effects; for instance, *Penocin A* disrupts the integrity of pathogen cell membranes, whereas *Enterolysin A* functions through the site-specific hydrolysis of cell wall peptidoglycan [35,36]. Therefore, these genomic features may provide a possible explanation for the stronger inhibitory activity of BGI-N8. However, because the antibacterial assay was performed using crude culture supernatants without pH neutralization, catalase treatment, protease treatment, heat treatment, or overlay/spot-on-lawn validation, the observed inhibition cannot be attributed specifically to bacteriocins. Organic acids, hydrogen peroxide, bacteriocin-like substances, or other extracellular metabolites may all contribute to the inhibitory activity. Thus, the antimicrobial results should be interpreted as preliminary in vitro evidence of extracellular inhibitory potential rather than proof of a defined antibacterial mechanism. Further mechanistic assays are needed to characterize the antimicrobial substances involved and to evaluate their potential relevance to microbiota-associated inflammation and metabolic health.

Colonization is required for probiotics to exert sustained host benefits. Both strains encode the *SecA2* transport system, which exports large serine-rich surface adhesins critical for initial host tissue attachment [37]. This *SecA2* system works with the *dltA/dltC* operon, which mediates the D-alanylation of lipoteichoic acids (LTAs) [38]. This modification reduces the net negative charge of the bacterial cell wall, thereby minimizing electrostatic repulsion and enhancing interactions with the negatively charged host mucosal surface [39]. Furthermore, the presence of “moonlighting” proteins, including enolase (*Eno*) [40], *GroEL* [41], and *DnaK* [42], indicates an additional layer of adhesive plasticity, as these proteins can function as high-affinity receptors for host plasminogen and fibronectin. BGI-N9 uniquely encodes L-fucose mutarotase (*FUOM*), which possibly interconverts L-fucose anomers, a sugar abundant in host mucin [43]. This trait may allow BGI-N9 to better utilize host-derived glycans, giving it a competitive advantage in the mucin-rich gut environment [44]. In vitro assays showed both strains had >70% auto-aggregation within 24 h, which may correlate with enhanced biofilm formation and gut persistence [45]. Building upon this, the adhesion indices of both strains are comparable to those of the commercial strain *L. paracasei* ATCC 334. These results suggest the adhesion potential of BGI-N8 and BGI-N9.

Metabolic syndrome is a major global health challenge, and gut microbiota dysbiosis is a key contributing factor. Probiotic modulation of the gut microbiota has therefore been proposed as a potential strategy for metabolic health support [32,46]. Targeted probiotics can improve energy homeostasis, lipid metabolism, and systemic immunity, alleviating metabolic syndrome symptoms [47,48]. BGI-N8 and BGI-N9 showed in vitro initial potential related to glucose and lipid homeostasis, supporting their preliminary functional indications. In terms of glucose-metabolism-related activity, both strains showed α-glucosidase inhibitory activity, with BGI-N8 achieving to 44.53%. This effect may be related to α-glucosidase inhibitors such as acarbose, which delay carbohydrate hydrolysis and reduce postprandial glucose spikes, reducing pancreatic β-cells’ burden [49]. However, whether BGI-N8 or BGI-N9 can produce comparable physiological effects requires further validation in animal or clinical studies Genomic analysis showed that BGI-N8 encodes a putative biosynthetic gene cluster containing a *HMG-CoA* synthase gene (ctg1_816), which may be associated with terpenoid-related biosynthetic potential [50]. Since some microbial terpenoids or natural terpenoid compounds have been reported as potential α-glucosidase inhibitors [51], this may provide a hypothesis for future mechanistic investigation. Beyond direct enzymatic inhibition, genomic analysis suggests that both strains possess acetate-related metabolic potential. Acetate is an important short-chain fatty acid SCFA, and previous studies have reported that SCFAs may participate in host metabolic regulation through receptors such as *GPR43* which possibly enhance insulin sensitivity and suppress adipose lipolysis [52,53,54]. This suggests the strains may exert synergistic hypoglycemic effects through direct enzymatic inhibition and systemic SCFA signaling. Nevertheless, as acetate production and *GPR43*-mediated host responses were not directly measured in this study, these genomic features cannot be considered evidence of systemic metabolic regulation. Instead, they provide a mechanistic hypothesis that may help guide future validation of the glucose-metabolism-related potential of BGI-N8 and BGI-N9.

Regarding lipid metabolism, both strains showed cholesterol-removal capacity in vitro, with BGI-N9 reaching a reduction rate of 74.49%. Genomic annotation identified bile salt hydrolase-related genes in both strains, suggesting a possible genetic basis for bile acid metabolism-related functions. First, acetate production may enhance lipid oxidation and reduce circulating free fatty acids [52,53]. Genome comparison revealed that BGI-N9 has a unique *fruR* regulator, which may optimize carbon flux and increase acetate production in the gut. Second, both strains encode the *bsh* gene, which deconjugates bile salts to prevent enterohepatic reabsorption and increase hepatic cholesterol utilization for bile acid synthesis [2]. The effectiveness of bsh depends on the bacterial count. In comparison, BGI-N9 has complete *dltA-D* operon and a unique ability to utilize FUOM, which means it may colonize more stably and survive longer in the gut, resulting in a stronger overall lipid-lowering effect. Both strains demonstrated exceptional cholesterol-lowering capacities in vitro, with BGI-N9 achieving a 74.49% reduction [55]. These genomic and phenotypic results demonstrate that BGI-N8 and BGI-N9 are promising probiotic candidates for metabolic health.

In conclusion, the present study aimed to perform genome-guided and in vitro phenotypic characterization of *Pediococcus pentosaceus* BGI-N8 and *Pediococcus acidilactici* BGI-N9 as candidate probiotic strains, focusing on genome-based safety screening, gastrointestinal tolerance, adhesion-related properties, crude supernatant antimicrobial activity, and glucose- and lipid-metabolism-related in vitro potential. However, the current evidence is limited to genome-based predictions and in vitro functional assays. Future studies, including targeted metabolite measurements, mechanistic validation, and in vivo experiments, are required to determine whether these strains can modulate glucose or lipid metabolism under physiological conditions. Furthermore, the synergistic effects of co-administering these two strains are to determine if their complementary functions provide enhanced benefits for obesity and type 2 diabetes management.

## Figures and Tables

**Table 1:** Information about the genome of BGI-N8 and BGI-N9.

Name	BGI-N8	BGI-N9
Bases	1,733,038	2,085,606
GC_Content	0.37	0.42
CDS	1698	1956
Contigs	1	1
rRNA	15	15
tRNA	56	57
tmRNA	1	1
Completeness	100.0%	100.0%
Contamination	0.08%	0.48%

## Data Availability

The data that support the findings of this study have been deposited into CNGBdb with accession number CNP0009405 (https://db.cngb.org/data_resources/project/CNP0009405; accessed on 1 July 2026).

## Supplementary Materials

The following supporting information can be downloaded at: https://www.mdpi.com/article/10.3390/microorganisms14081614/s1, Table S1: BGI-N8 and BGI-N9-related carbon source utilization genes; Table S2: Carbon source utilization of BGI-N8 and BGI-N9; Table S3: Virulence-associated genes identified in BGI-N8 and BGI-N9; Table S4: Antibiotic resistance genes in BGI-N8 and BGI-N9; Table S5: Gene function annotation of genes related to digestive tract adaptation in BGI-N8 and BGI-N9; Table S6: Comparative analysis of adhesion-associated genes in BGI-N8 and BGI-N9; Table S7: Functional gene annotation of hypoglycemic and hypolipidemic metabolic pathways in BGI-N8 and BGI-N9.

## Abbreviations

The following are used in this manuscript:

Abbreviation	Full name
ANI	Average Nucleotide Identity
BHI	Brain Heart Infusion
BSH	Bile salt hydrolase
CAZymes	Carbohydrate-active enzymes
CARD	Comprehensive Antibiotic Resistance Database
CGR2	Cultivated Genome Reference 2
COG	Clusters of Orthologous Groups
DMEM	Dulbecco’s Modified Eagle Medium
EC	Enzyme Commission
EDTA	Ethylenediaminetetraacetic acid
GTDB-Tk	Genome Taxonomy Database Toolkit
HMG-CoA	Hydroxymethylglutaryl-CoA
iTOL	Interactive Tree of Life
KEGG	Kyoto Encyclopedia of Genes and Genomes
LAB	Lactic acid bacteria
LPS	Lipopolysaccharide
MRS	De Man, Rogosa & Sharpe
NCBI	National Center for Biotechnology Information
OD	Optical density
PBS	Phosphate-buffered saline
PCR	Polymerase chain reaction
pNPG	p-nitrophenyl-α-D-glucopyranoside
RGI	Resistance Gene Identifier
RiPP	Ribosomally synthesized and post-translationally modified peptides
SCFA	Short-chain fatty acid
SD	Standard deviation
T3PKS	Type III Polyketide Synthase
VFDB	Virulence Factor Database
WGS	Whole-genome sequencing
