# Supplementary material for: Integrated Genomics and Phenotypic Analysis of Pediococcus pentosaceus BGI-N8 and Pediococcus acidilactici BGI-N9: Partial Evidence Suggesting In Vitro Probiotic Properties to Glycolipid Metabolism Regulation Potential
Source: Microorganisms. 2026 Jul 24;14(8):1614. doi: 10.3390/microorganisms14081614 (PMC13515514; doi:10.3390/microorganisms14081614)
Supplement: Supplementary file 1 [file microorganisms-14-01614-s001.zip › microorganisms-4381820-supplementary.pdf]

Supplementary material

Table S1. BGI-N8 and BGI-N9 related carbon source utilization genes.

query	Preferred_name	EC	PFAMs
BGI-N8_00135	galK D-Galactose	2.7.1.6	GHMP_kinases_C,GHMP_kinases_N,GalKase_gal_bdg
BGI-N8_00216	galR D-Galactose	-	LacI,Peripla_BP_3
BGI-N8_00222	galE D-Galactose	5.1.3.2	Epimerase,GDP_Man_Dehyd
BGI-N8_00223	galT D-Galactose	2.7.7.12	GalP_UDP_tr_C,GalP_UDP_transf
BGI-N8_00484	pgm D-Galactose	5.4.2.2	PGM_PMM_I,PGM_PMM_II,PGM_PMM_III,PGM_PMM_IV
BGI-N8_00728	glcK Glucose	2.7.1.2	ROK
BGI-N8_01281	glcU Glucose	-	Sugar_transport
BGI-N9_00430	galK D-Galactose	2.7.1.6	GHMP_kinases_C,GHMP_kinases_N,GalKase_gal_bdg
BGI-N9_00430	pgm D-Galactose	5.4.2.2	PGM_PMM_I,PGM_PMM_II,PGM_PMM_III,PGM_PMM_IV
BGI-N9_00732	galR D-Galactose	-	LacI,Peripla_BP_3
BGI-N9_00738	galE D-Galactose	5.1.3.2	Epimerase,GDP_Man_Dehyd
BGI-N9_00739	galT D-Galactose	2.7.7.12	GalP_UDP_tr_C,GalP_UDP_transf
BGI-N9_00812	glcK Glucose	2.7.1.2	ROK
BGI-N9_01454	glcU Glucose	-	Sugar_transport
BGI-N9_01271	fruA D-Fructose	2.7.1.202	PTS_EIIA_2,PTS_EIIC,PTS_IIB
BGI-N9_01273	fruR D-Fructose	-	DeoRC,HTH_DeoR

Table S2. Carbon source utilization of BGI-N8 and BGI-N9.

carbon source	BGI-N8	BGI-N9	carbon source	BGI-N8	BGI-N9
blank	-	-	Esculin	+	+
glycerol	-	-	salicin	+	±
PETN	-	-	cellobiose	+	+
D- arabinose	-	-	maltose	+	-
L- arabinose	+	+	lactose	±	-

ribose	+	+	melibiose	-	-
D-xylose	-	+	saccharose	-	-
L-xylose	-	-	D-Trehalose	+	+
adonite	-	-	Inulin	-	-
β-Methyl-D-xyloligoside	-	-	Trehalose	-	-
D-Galactose	+	+	raffinose	-	-
Glucose	+	+	starch	-	-
D-Fructose	+	+	glycogen	-	-
D-Mannose	+	+	xylitol	-	-
L-Sorbose	-	-	dextrin	±	±
rhamnose	±	±	D- turanose	-	-
dulcitol	-	-	D- lyxose	-	-
Inositol	-	-	D- tagatose	±	±
mannitol	-	-	sucrose	-	-
sorbic alcohol	-	-	L-Rhamnose	-	-
α-Methyl-D-mannoside	-	-	D-arabitol	-	-
α-Methyl-D-glucoside	-	-	L-arabitol	-	-
N-acetylglucamine	+	±	gluconate	-	-
amygdalin	+	±	2-keto-glucuronide	-	-
arbutin	+	-	5-ketogluconate	-	-

"+" indicates a positive result, "−" indicates a negative result, "±" indicates a weakly positive result.

Table S3 Virulence-associated genes identified in BGI-N8 and BGI-N9.

Strain	Accession.Version	Gene code	Gene name	Similarity	Molecular Function	Functional Category
BGI-N8	P.pentosaceus_BGI-N8_01357	VFG000079	*clpC*	55.788	ATP-binding subunit C of Clp endopeptidase	Stress Survival
	P.pentosaceus_BGI-N8_00604	VFG000080	*clpE*	59.811	ATP-dependent protease	Stress Survival
	P.pentosaceus_BGI-N8_00460	VFG012095	*groEL*	65.406	Chaperonin GroEL	Adhesion
	P.pentosaceus_BGI-N8_00709	VFG048830	*gndA*	67.872	NADP-dependent phosphogluconate dehydrogenase	Immunomodulation
	P.pentosaceus_BGI-N8_00460	VFG001855	*htpB*	56.952	Hsp60 heat shock protein HtpB	Adhesion
	P.pentosaceus_BGI-N8_01075	VFG046465	*tufA*	69.211	Elongation factor Tu	Adhesion
	P.pentosaceus_BGI-N8_00576	VFG002182	*cpsI*	60.27	UDP-galactopyranose mutase	Immunomodulation
	P.pentosaceus_BGI-N8_	VFG0013	*cps4I*	58.889	Capsular	Immunomodula

	00594	73			polysaccharide synthesis protein	tion
	P.pentosaceus_BGI-N8_00576	VFG001967	*glf*	57.373	UDP-galactopyranose mutase	Immunomodulation
	P.pentosaceus_BGI-N8_00594	VFG001312	*cap8P*	56.474	Type 8 capsular polysaccharide synthesis protein Cap8P	Immunomodulation
	P.pentosaceus_BGI-N8_00482	VFG000964	*hasC*	69.932	UTP--glucose-1-phosphate uridylyltransferase HasC	Immunomodulation
	P.pentosaceus_BGI-N8_01031	VFG001405	*sigA/rpoV*	62.706	RNA polymerase sigma factor SigA	Regulation
	P.pentosaceus_BGI-N8_00868	VFG002190	*cpsA/uppS*	62.712	Undecaprenyl diphosphate synthase	Immunomodulation
	P.pentosaceus_BGI-N8_00493	VFG000077	*clpP*	71.066	Proteolytic subunit of ATP-dependent Clp protease	Stress Survival
	P.pentosaceus_BGI-N8_01357	VFG002076	*clpV1*	50.388	Type VI secretion system AAA+ family ATPase	Effector Delivery System
	P.pentosaceus_BGI-N8_00869	VFG002189	*cpsB/cdsA*	50.562	Phosphatidate cytidylyltransferase	Immunomodulation
	P.pentosaceus_BGI-N8_00949	VFG037100	*msrA/BpilB*	64.234	Methionine sulfoxide reductase A/B	Stress Survival
	P.pentosaceus_BGI-N8_00572	VFG001937	*Cj1136*	51.765	Glycosyltransferase	Immunomodulation
BGI-N9	P.acidilactici_BGI-N9_00406	VFG012095	*groEL*	66.163	Chaperonin GroEL	Adhesion
	P.acidilactici_BGI-N9_00428	VFG000964	*hasC*	70.169	UTP--glucose-1-phosphate uridylyltransferase HasC	Immunomodulation
	P.acidilactici_BGI-N9_00439	VFG000077	*clpP*	71.574	Proteolytic subunit of ATP-dependent Clp protease	Stress Survival
	P.acidilactici_BGI-N9_00596	VFG000080	*clpE*	60.768	ATP-dependent protease	Stress Survival
	P.acidilactici_BGI-N9_00791	VFG048830	*gndA*	67.66	NADP-dependent phosphogluconate	Immunomodulation

				dehydrogenase	
P.acidilactici_BGI-N9_00900	VFG002190	*cpsA/uppS*	63.983	Undecaprenyl diphosphate synthase	Immunomodulation
P.acidilactici_BGI-N9_00986	VFG037100	*msrA/BpilB*	60	Methionine sulfoxide reductase A/B	Stress Survival
P.acidilactici_BGI-N9_01113	VFG001405	*sigA/rpoV*	63.036	RNA polymerase sigma factor SigA	Regulation
P.acidilactici_BGI-N9_01250	VFG046465	*tufA*	69.975	Elongation factor Tu	Adhesion

Table S4 Antibiotic Resistance Genes in BGI-N8 and BGI-N9.

Strain	Gene name	RGI criteria	Drug resistance category	Resistance mechanism	Similarity (%)	Coverage (%)
BGI-N8	*vanT*	Strict	glycopeptide antibiotics	alteration of antibiotic targets	36.53	75.88
	sdrM	Strict	Fluoroquinolone antibiotics, disinfectants	antibiotic efflux	34.81	109.4
BGI-N9	*vanT*	Strict	glycopeptide antibiotics	alteration of antibiotic targets	31.42	52.67
	qacG	Strict	disinfectant	antibiotic efflux	48.11	99.07

Table S5. Gene function annotation of genes related to digestive tract adaptation in BGI-N8 and BGI-N9

Types	Query	Preferred _name	EC	KEGG	PFAMs
Gut persistence	N8_00291	*celC*	2.7.1.196,2.7.1.205	K02759	PTS_IIA
	N9_00224	*celC*	2.7.1.196, 2.7.1.205	K02759	PTS_IIA
	N9_00277	*pepO*	-	K07386	Peptidase_M13, Peptidase_M13_N
	N9_00865	*plsC*	2.3.1.51	K00655	Acyltransferase
	N9_01086	*pyk*	2.7.1.40	K00873	PEP-utilizers, PK, PK_C
	N9_01818	*eno*	4.2.1.11	K01689	Enolase_C, Enolase_N
	N9_00545	*epsB*	-	-	GNVR, Wzz
	N9_01679	*srtA*	3.4.22.70	K07284	Sortase
	N9_01699	*dltD*	-	K03740	DltD
	N9_01700	*dltC*	6.1.1.13	K14188	PP-binding
	N9_01701	*dltB*	-	K03739	MBOAT
Acid resistance	N8_00860	*plsC*	2.3.1.51	K00655	Acyltransferase
	N8_01024	*pyk*	2.7.1.40	K00873	PEP-utilizers, PK,PK_C
	N8_01247	*atpC*	-	K02114	ATP-synt_DE, ATP-synt_DE_N
	N8_01248	*atpD*	3.6.3.14	K02112	ATP-synt_ab, ATP-synt_ab_N
	N8_01249	*atpG*	-	K02115	ATP-synt
	N8_01250	*atpA*	3.6.3.14	K02111	ATP-synt_ab, ATP-synt_ab_C, ATP-synt_ab_N
	N8_01251	*atpH*	-	K02113	OSCP
	N8_01252	*atpF*	-	K02109	ATP-synt_B

	N8_01253	*atpE*	-	K02110	ATP-synt_C
	N8_01254	*atpB*	-	K02108	ATP-synt_A
	N8_01589	*asp2*	-		Asp23
	N9_01702	*dltA*	6.1.1.13	K03367	AMP-binding, AMP-binding_C
	N9_00437	*clpP*	3.4.21.92	K01358	CLP_protease
	N9_00443	*eno*	4.2.1.11	K01689	Enolase_C, Enolase_N
	N9_00594	*clpE*	-	K03697	AAA, AAA_2, ClpB_D2-small, UVR
	N9_00887	*grpE*	-	K03687	GrpE
	N9_00888	*dnaK*	-	K04043	HSP70
	N9_00889	*dnaJ*	-	K03686	DnaJ, DnaJ_C, DnaJ_CXXCXGXG
	N9_00960	*msrB*	1.8.4.12	K07305	SelR
	N9_00972	*hslU*	-	K03667	AAA_2, ClpB_D2-small
	N9_00973	*hslV*	3.4.25.2	K01419	Proteasome
	N9_01090	*clpB*	-	K03695	AAA, AAA_2, ClpB_D2-small, Clp_N
	N9_01223	*clpX*	-	K03544	AAA_2, ClpB_D2-small, zf-C4_ClpX
	N9_01260	*cspA*	-	K03704	CSD
	N9_01559	*clpC*	-	K03696	AAA, AAA_2, ClpB_D2-small, Clp_N, UVR
	N9_01571	*cspC*	-	K03704	CSD
	N9_01656	*hslO*	-	K04083	HSP33
	N9_01384	*atpC*	-	K02114	ATP-synt_DE, ATP-synt_DE_N
	N9_01385	*atpD*	3.6.3.14	K02112	ATP-synt_ab, ATP-synt_ab_N
	N9_01386	*atpG*	-	K02115	ATP-synt
	N9_01387	*atpA*	3.6.3.14	K02111	ATP-synt_ab, ATP-synt_ab_C, ATP-synt_ab_N
	N9_01388	*atpH*	-	K02113	OSCP
	N9_01389	*atpF*	-	K02109	ATP-synt_B
	N9_01390	*atpE*	-	K02110	ATP-synt_C
	N9_01391	*atpB*	-	K02108	ATP-synt_A
	N9_01430	*asp2*	-	K12269	Asp2
Bile resistance	N8_00340	*pepO*	-	K07386	Peptidase_M13, Peptidase_M13_N
	N8_00434	*oppA*	-	K15580	SBP_bac_5
	N8_00435	*oppB*		K15581	BPD_transp_1
	N8_00436	*oppC*	-	K15582	BPD_transp_1,

				OppC_N
N8_00437	*oppD*	-	K15583	ABC_tran,oligo_HPY
N8_00438	*oppF*	-	K10823	ABC_tran,oligo_HPY
N8_00576	*glf*	5.4.99.9	K01854	GLF,NAD_binding_8
N8_00696	*rpmI*	-	K02916	Ribosomal_L35p
N8_00697	*rplT*	-	K02887	Ribosomal_L20
N8_00708	*rpmF*	-	K02911	Ribosomal_L32p
N8_00735	*glnA*	6.3.1.2	K01915	Gln-synt_C,Gln-synt_N
N8_00744	*rplU*	-	K02888	Ribosomal_L21p
N8_00746	*rpmA*	-	K02899	Ribosomal_L27
N8_00769	*rpmB*	-	K02902	Ribosomal_L28
N8_00780	*rpsP*	-	K02959	Ribosomal_S16
N8_00783	*rplS*	-	K02884	Ribosomal_L19
N8_00864	*rpsB*	-	K02967	Ribosomal_S2
N8_00951	*ppaC*	3.6.1.1	K15986	DHH,DHHA2
N8_01011	*rpsA*	-	K02945	S1
N8_01041	*rpsU*	-	K02970	Ribosomal_S21
N8_01078	*rpsO*	-	K02956	Ribosomal_S15
N8_01079	*rpsT*	-	K02968	Ribosomal_S20p
N8_01228	*rpsD*	-	K02986	Ribosomal_S4,S4
N8_01418	*rplL*	-	-	Ribosomal_L12, Ribosomal_L12_N
N8_01512	*oppA*	-	-	SBP_bac_5
N8_01630	*cfa*	-	-	CMAS
N9_01778	*asp2*	-	-	Asp23
N9_00048	*mntH*	-	K03322	Nramp
N9_00197	*proV*	-	K05847	ABC_tran
N9_00227	*nox*	1.6.3.4	K17869	Pyr_redox_2, Pyr_redox_dim
N9_00350	*corA*	-	K03284	CorA
N9_00352	*trxA*	-	K03671	Thioredoxin
N9_00427	*trxB*	1.8.1.9	K00384	Pyr_redox_2
N9_00929	*lspA*	3.4.23.36	K03101	Peptidase_A8
N9_00959	*msrA*	1.8.4.11, 1.8.4.12	K07304, K12267	PMSR, SelR
N9_00981	*npr*	1.11.1.1	K05910	Pyr_redox_2, Pyr_redox_dim
N9_01206	*katA*	1.11.1.6	K03781	Catalase, Catalase-rel
N9_01325	*trxA*	-	K03671	Thioredoxin
N9_01360	*tpx*	1.11.1.15	K11065	AhpC-TSA, Redoxin
N9_01606	*nrdH*	-	K06191	Glutaredoxin

N9_01752	*nox*	-	-	Pyr_redox_2, Pyr_redox_dim
N9_01804	*mntH*	-	K03322	Nramp
N9_01820	*cfa*	2.1.1.79	K00574	CMAS
N9_00753	*rpmI*	-	K02916	Ribosomal_L35p
N9_00754	*rplT*	-	K02887	Ribosomal_L20
N9_00764	*rpmF*	-	K02911	Ribosomal_L32p
N9_00793	*glnA*	6.3.1.2	K01915	Gln-synt_C, Gln-synt_N
N9_00801	*rplU*	-	K02888	Ribosomal_L21p
N9_00803	*rpmA*	-	K02899	Ribosomal_L27
N9_00826	*rpmB*	-	K02902	Ribosomal_L28
N9_00837	*rpsP*	-	K02959	Ribosomal_S16
N9_00842	*rplS*	-	K02884	Ribosomal_L19
N9_00869	*rpsB*	-	K02967	Ribosomal_S2
N9_00964	*ppaC*	3.6.1.1	K15986	DHH, DHHA2
N9_01074	*rpsA*	-	K02945	S1
N9_01086	*pyk*	2.7.1.40	K00873	PEP-utilizers, PK, PK_C
N9_01228	*rpsO*	-	K02956	Ribosomal_S15
N9_01229	*rpsT*	-	K02968	Ribosomal_S20p
N9_01365	*rpsD*	-	K02986	Ribosomal_S4, S4
N9_01609	*rplL*	-	K02935	Ribosomal_L12, Ribosomal_L12_N

Table S6 Comparative analysis of adhesion-associated genes in BGI-N8 and BGI-N9

Strain Gene ID	Uniprot ID	Gene Name	Function Description
BGI-N8_00226	P23284	*PPIB*	PPIase (cyclophilin-type); catalyzes proline imidic peptide bond cis-trans isomerization to assist protein folding.
BGI-N8_00302	P9WMN3	*glmU*	Bifunctional enzyme; catalyzes last two steps in de novo UDP-N-acetylglucosamine biosynthesis.
BGI-N8_00340	P42359	-	Belongs to peptidase M13 family.
BGI-N8_00416	Q6IMN8	*alrA*	Aldo/keto reductase; catalyzes NADPH-dependent carbonyl compound reduction.
BGI-N8_00460	Q9KKF0	*groEL*	HSP60 family chaperonin; forms 14-subunit complex with GroES to assist protein folding.
BGI-N8_00467	Q97P83	*secA2*	Accessory Sec translocase ATPase; involved in protein export and virulence.
BGI-N8_00481	Q93D83	*gpsA*	NAD-dependent glycerol-3-phosphate dehydrogenase; key enzyme in phospholipid synthesis.
BGI-N8_00497	P47543	*gapA*	Glyceraldehyde-3-phosphate dehydrogenase; key glycolytic enzyme.
BGI-N8_00499	Q96VN5	*TPI1*	Triosephosphate isomerase; catalyzes triosephosphate isomerization in glycolysis.
BGI-N8_00500	Q97QS2	*eno*	Enolase; glycolytic enzyme. Also functions as a plasminogen receptor (moonlighting function).
BGI-N8_00754	A0A0H3PEK7	*tlyA*	TlyA family protein; catalyzes 23S rRNA methylation,

			participates in biofilm formation.
BGI-N8_00857	Q93SM0	*lexA*	Repressor of the SOS response to DNA damage.
BGI-N8_00883	P0A3J0	*dnaK*	Molecular chaperone (HSP70 family); acts as chaperone under stress conditions.
BGI-N8_00940	A4VWG9	*rqcH*	Ribosome quality control factor; mediates degradation of stalled nascent chains.
BGI-N8_00951	P95765	*ppaC*	Inorganic pyrophosphatase; belongs to PPase class C family.
BGI-N8_00989	P22392	*NME2*	Nucleoside diphosphate kinase; catalyzes NTP synthesis.
BGI-N8_00999	Q93DA2	*metN*	ATP-binding component of an ABC transporter for methionine import.
BGI-N8_01075	Q74JU6	*tuf*	Elongation factor Tu; delivers aminoacyl-tRNA to the ribosome during protein synthesis.
BGI-N8_01207	P68845	*recA*	Key enzyme in DNA repair and homologous recombination.
BGI-N8_01248	P56480	*Atp5f1b*	ATP synthase subunit beta; catalytic component of ATP synthase complex.
BGI-N8_01438	P82612	*GPM1*	Phosphoglycerate mutase; glycolytic enzyme with antigenic properties.
BGI-N8_01492	Q93D93	*htpX*	Membrane-associated protease (Peptidase M48B family).
BGI-N8_01516	Q9S389	*dltC*	D-alanyl carrier protein for lipoteichoic acid alanylation.
BGI-N8_01518	Q8Y8D4	*dltA*	D-alanine--D-alanyl carrier protein ligase for cell wall modification.
BGI-N8_01641	Q840P8	*saeR*	Response regulator of SaeRS; regulates virulence factors.
BGI-N8_01648	Q93D95	*rsmG*	rRNA methyltransferase; specifically methylates 16S rRNA.
BGI-N8_01721	Q6GGZ3	*arlR*	Response regulator of ArlSR; regulates adhesion and autolysis.
BGI-N8_01768	Q8Y3I2	*yidC2*	Membrane protein insertase; mediates protein insertion and folding.
BGI-N9_00161	Q5R8S7	*PPIA*	Peptidyl-prolyl cis-trans isomerase; molecular chaperone.
BGI-N9_00242	P9WMN3	*glmU*	Bifunctional enzyme for UDP-N-acetylglucosamine biosynthesis.
BGI-N9_00282	P42359	-	Peptidase (M13 family).
BGI-N9_00361	Q6IMN8	*alrA*	Aldo/keto reductase; NADPH-dependent reduction of carbonyl compounds.
BGI-N9_00406	Q9KKF0	*groEL*	Molecular chaperone; essential for protein folding (HSP60 family).
BGI-N9_00413	Q97P83	*secA2*	Accessory Sec translocase ATPase; involved in protein export.
BGI-N9_00415	Q6GGZ3	*arlR*	Response regulator; regulates adhesion, autolysis, and virulence.
BGI-N9_00427	Q93D83	*gpsA*	Glycerol-3-phosphate dehydrogenase; phospholipid synthesis.
BGI-N9_00446	Q97QS2	*eno*	Enolase; glycolytic enzyme and plasminogen receptor.
BGI-N9_00697	A2VDF0	*FUOM*	L-fucose mutarotase; involved in host mucin utilization.
BGI-N9_00915	P0A3J0	*dnaK*	Molecular chaperone (HSP70 family).
BGI-N9_01751	Q9S389	*dltC*	D-alanyl carrier protein for lipoteichoic acid alanylation.
BGI-N9_01753	Q8Y8D4	*dltA*	D-alanine--D-alanyl carrier protein ligase.

Table S7 Functional gene annotation of hypoglycemic and hypolipidemic Metabolic Pathways in BGI-N8 and BGI-N9

Metabolism pathway	Gene	EC code	Eczymed Encoded	N8 quantities	N9 quantities	Primary reaction	Functional role
Acrylate pathway	*ldh*	1.1.1.27	Lactate dehydrogenase	2	2	Pyruvate ↔ Lactate	Regenerates NAD+ and controls the redox balance. Serves as a potential precursor for the acrylate pathway, which redirects lactate to the short-chain fatty acid propionate, a known modulator of host lipid metabolism.
Bile salt hydrolase / Choloylglycine hydrolase	*bsh*	3.5.1.24	Bile salt hydrolase	1	1	Conjugated bile acid → Deconjugated bile acid + AA	The key enzyme for bile acid deconjugation. Deconjugated bile acids are less efficiently reabsorbed, leading to increased hepatic cholesterol consumption for de novobile acid synthesis, thereby reducing serum cholesterol levels.
Acetate Generation Pathway	*zwf*	1.1.1.363,1.1.1.49	Glucose-6-phosphate dehydrogenase	1	1	G6P → 6PGL	Initiates the oxidative phase of the pentose phosphate pathway (PPP), generating reducing power in the form of NADPH. This is often the rate-limiting step.
	pgl	3.1.1.31	6-Phosphogluconolactonase	1	1	6PGL → 6PG	Hydrolyzes the unstable lactone (6PGL) to 6-phosphogluconate (6PG), facilitating the subsequent oxidative decarboxylation step.
	gnd	1.1.1.343,1.1.1.44	6-Phosphogluconate dehydrogenase	1	1	6PG → Ru5P + CO_2	Catalyzes the oxidative decarboxylation that produces pentose sugar (ribulose-5-phosphate, Ru5P), CO_2, and a second molecule of NADPH.
	rpe	5.1.3.1	Ribulose-5-phosphate epimerase	1	1	Ru5P ↔ Xu5P	Interconverts pentose phosphates to provide essential substrates, primarily xylulose-5-phosphate (Xu5P), for the phosphoketolase reaction.
	xfp	4.1.2.22,4.1.2.9	Phosphoketolase	1	1	Xu5P + P_i → Acetyl-P + G3P	A key branch-point enzyme that cleaves xylulose-5-phosphate to directly generate acetyl-phosphate (Acetyl-P), the immediate precursor to acetate, and glyceraldehyde-3-phosphate (G3P).
	acyP	3.6.1.7	Acylphosphatase	1	1	Acetyl-P → Acetate	Catalyzes the hydrolytic dephosphorylation of acetyl phosphate, resulting in the final production and excretion of acetate.
